# Evaluating Methodological Coherence and Evidence Recognition in Digital Health Systematic Reviews: Sample-based Meta-research Study

**DOI:** 10.2196/78210

**Published:** 2026-04-16

**Authors:** Uwe Buddrus, Jan-David Liebe

**Affiliations:** 1 Professorship Digital Society, Faculty of Business, Economics and Social Sciences, Hochschule Osnabrück, CO-Gebäude, Raum 102, Albrechtstraße 30 a, Osnabrück, 49076, Germany, 49 541 969 ext 7019; 2 Institute of Medical Informatics, UMIT - Private Universität für Gesundheitswissenschaften, Medizinische Informatik und Technik, Hall, Tyrol, Austria

**Keywords:** systematic reviews, digital health interventions, evidence, outcomes, effects, PICO, title and abstract screening, evidence recognition, methodological flaws, population or problem, intervention, comparison, and outcome

## Abstract

**Background:**

Despite a growing number of systematic reviews on digital health interventions, many do not sufficiently support the recognition of conclusive evidence. Methodological shortcomings may impede the identification and communication of robust findings. Abstracts are the basis for study selection in systematic reviews and are increasingly used in automated screening processes and rapid assessments.

**Objective:**

This meta-research study examines to what extent systematic reviews apply methodological standards—particularly the specification of PICO (population or problem, intervention, comparison, and outcome) elements—and how this relates to the likelihood of conclusive evidence recognition. It is based on a random sample and focuses on the assessment at the abstract level, as abstracts are used independently of the specific review choice to screen and select studies for evidence synthesis, making them critical for evidence recognition.

**Methods:**

Following PRISMA (Preferred Reporting Items for Systematic Reviews and Meta-Analyses) guidelines, we conducted a comprehensive database search (2011‐2023). From 2528 eligible systematic reviews, a random sample of 250 abstracts was analyzed descriptively. Abstracts were assessed for PICO specification and evidence conclusiveness in the context of further study characteristics.

**Results:**

In total, 48% (119/250) of reviews showed low or very low PICO specification, and 64% (159/250) reported inconclusive or weak evidence. Higher specification of outcomes and problems was moderately associated with conclusive evidence. Beside the formulation of the research question along the PICO scheme, we identified recurring issues in search and screening strategy design (eg, limited database use, vague search terms, and long search periods), restrictive eligibility criteria (eg, exclusive reliance on randomized controlled trials), inconsistent use of quality appraisal tools, and underusage of alternative synthesis methods to hinder evidence recognition.

**Conclusions:**

Our findings suggest that methodological coherence across all review stages is a necessary condition to ensure conclusions and evidence-informed decisions in the digitalization of health care are both valid and meaningful. A structured PICO-based framework, which is aligned with current research, builds on well-established categories and provides clear and differentiated definitions that may enhance the focus and evidentiary strength of future reviews.

## Introduction

The digital transformation of health care has led to a substantial increase in studies evaluating the effects of digital health interventions (DHIs) [1,2]. Despite this growing body of research, decision-makers still lack conclusive and consistent evidence on the effects of DHIs. Systematic reviews, although considered the gold standard for evidence synthesis, frequently fail to provide robust conclusions. This discrepancy raises fundamental questions about the methodological quality and scientific rigor of such reviews. Given the role of systematic reviews in shaping digital health policy and clinical guidance, it is crucial to understand how methodological shortcomings affect the reliability of their conclusions.

Poorly formulated research questions and ineffective evidence search and screening strategies were previously identified to impede the retrieval and synthesis of relevant evidence [3].

The PICO (population, problem, or patient, intervention, comparison, and outcome) framework has been widely adopted in clinical research to formulate answerable questions and design efficient search strategies [3-6]. Empirical studies confirm that applying PICO is predominant in clinical practice for structuring questions that compare one intervention with an alternative [7], enhances the precision of literature searches, and supports the identification of relevant studies [8]. Using PICO is recommended for interventional effectiveness and economic cost-effectiveness studies [6], especially pertinent for analyzing DHI effects.

It is evident that PICO is critical in the research process, including the formulation of the research questions, keyword development for the search strategy, and the definition of eligibility criteria. Nonetheless, its application in systematic reviews appears to be inconsistent and often not rigorous in these early stages of the research process. This may limit the capacity of systematic reviews to deliver actionable conclusions, especially in a field as heterogeneous and dynamic as digital health. Thus, we consider PICO as a methodological key factor and hypothesize that low PICO specification correlates with low evidence recognition.

To date, no comprehensive analysis has quantified the extent and specificity of the use of PICO elements in systematic reviews of DHIs. Furthermore, the relationship between the degree of PICO specification and the likelihood of finding conclusive evidence remains underexplored.

To address these gaps, this study investigates the extent to which systematic reviews on DHIs specify PICO elements and examines whether this level of specification is associated with the likelihood of reporting conclusive evidence.

Given that abstracts are the basis for study selection in systematic reviews and are increasingly used in automated screening processes and rapid assessments, abstracts serve as a primary information source for evidence identification. This study, therefore, focuses on the abstract level to evaluate how methodological structure is communicated and how it may influence the perceived strength of evidence when screening and selecting studies for full-text review.

The following research questions guided our analysis:

1. How frequently and to what degree are the PICO elements specified in the abstracts of systematic reviews and meta-analyses on DHI effects?
2. What is the relationship between the specification of PICO elements and the availability of conclusive evidence?
3. How are methodological study characteristics (eg, search period, number of databases searched, included study types, and number of included studies) associated with the likelihood of conclusive evidence in systematic reviews on DHIs?

## Methods

### Overview

We conducted this meta-research study following selected principles of the scoping review methodology according to PRISMA-ScR (Preferred Reporting Items for Systematic Reviews and Meta-Analyses extension for Scoping Reviews) [9], for example, broad inclusion criteria and descriptive synthesis. We performed a structured, quantitative content analysis on a random sample of 250 abstracts from systematic reviews and meta-analyses researching the effects of DHIs.

Table 1 provides an overview of the key aspects in each methodological step.

**Table 1.** Summary overview of methodological steps.

Methodological step	Description
Search strategy	Systematic reviews or meta-analyses A broad range of DHIs^a^ A broad range of outcomes In any health care setting From 2011 to October 2023 In 5 leading databases
Study selection	Title and abstract screening in reviewer pairs 10 inclusion and 6 exclusion criteria Random sample of 250 abstracts for 95% CI and 6% margin of error
Data extraction	Exclusively from titles and abstracts 16 data extraction fields and variables
Data analysis	Transformation into metric, nominal, and ordinal variables Univariate quantitative descriptive analysis of study characteristics, and representation of PICO^b^ elements and criteria for conclusive evidence Bivariate and correlation analysis using Spearman rank correlation coefficient (ρ) of PICO specification and evidence

a DHI: digital health intervention.

b PICO: population or problem, intervention, comparison, and outcome.

### Search Strategy

The search strategy was developed with the aim to include existing systematic reviews and meta-analyses which address (1) a broad range of DHI technologies and (2) a broad range of potential associated outcomes in (3) any health care setting.

A total of 33 DHI-related and 41 outcome-related search terms were used and—depending on the database searched—applied as Medical Subject Headings terms to subject headings or as plain search terms on titles and abstracts. A complete documentation of all search terms is available in Multimedia Appendix 1.

A comprehensive literature search was conducted for the time frame 2011 to October 2021 for a preceding umbrella review project, which was updated in October 2023 for more in-depth research.

To capture a large heterogeneity of care settings and DHI application scenarios, the search included all areas of health care—from inpatient hospital care through outpatient and community-based care to self-care, without any geographic boundaries.

The search was limited to systematic reviews or meta-analyses published in English or German.

We searched 5 leading databases—Scopus, AISeL, EBSCO or CINAHL, Cochrane Library, and MEDLINE via PubMed.

### Study Selection

The search results were uploaded to Covidence (Veritas Health Innovation Ltd), a software for managing and streamlining systematic reviews. All potential studies for further analyses, such as evidence and gap mapping, were screened in pairs by UB and Jan-Oliver Kutza, and Johannes Thye and Moritz Esdar, and discrepancies were resolved through discussion or consultation with a senior reviewer (JDL or Ursula Hübner). A complete documentation of all inclusion and exclusion criteria is available in Multimedia Appendix 2.

The initial abstract review and screening process was completed in February 2024. After all abstracts were screened, 2528 eligible studies for subsequent analyses were exported to Microsoft Excel, additional duplicates were identified, checked, and omitted, and a random sample of 250 studies was drawn, using the random number assignment function in Excel, by the lead author (UB). The sample size of 250 abstracts was chosen as the basis for the detailed data extraction and quantitative analysis, to represent about 10% (250/2528) of eligible studies, and to have a 95% confidence level that the actual value is within ±6% of the measured or surveyed value when exploring potential relationships between study characteristics reported in the abstracts and the likelihood to find conclusive evidence in the full texts.

### Data Extraction

In line with our research questions, a set of 16 data extraction fields and variables was developed. Next to meta-information on study design (particularly if meta-analysis was performed and randomized controlled trial [RCT]–focus), publication year, search period in years, databases searched, the total number of studies identified by the search strategy, the number of included studies, the journal, and statements regarding evidence quality and quality assessment tools, these were the descriptions related to the PICO elements problem and population, intervention, comparator, and outcome, as well as the results and conclusions, representing the scope of evidence.

All information was exclusively identified and extracted from titles and abstracts.

This decision was based on the relevance of abstracts as the main communicative interface in the screening process for evidence synthesis and decision-making contexts. Abstracts are increasingly used in automated screening and mapping tools, making them a critical unit of analysis.

### Data Analysis

#### Data Transformation and Classification

The quantitative analysis focused on the meta-information of study characteristics and the representation of PICO elements and criteria for conclusive evidence in abstracts of systematic reviews and meta-analyses. To facilitate quantification, extracted data were transformed into metric (eg, search period in year, number of databases searched, and cumulative PICO score), nominal (eg, yes or no for meta-analysis and RCT-focus), and ordinal variables (eg, search period: short=1‐10 y, medium=11‐20 y, and long=21+ y; number of databases: high=7+, medium=4‐6, and low<4; and number of studies included: high=21+, medium=11‐20, and low=1‐10).

The ordinal classification scheme for PICO elements and likelihood of conclusive evidence was developed through multiple iterations, based on a qualitative review of the wording used in titles, objectives, methods, results, and conclusions of the abstracts. Additionally, the classification of the likelihood of conclusive evidence was informed by literature. The criteria were refined until they could be unambiguously applied to every abstract in the sample. The resulting ordinal variables for the classification of PICO elements were high=3, medium=2, low=1, and not specified=0, and for the likelihood of conclusive evidence high=3, medium=2, low=1, and inconclusive=0. All criteria accompanied by examples from extracted data are available in the Multimedia Appendix 3. To ensure clarity, consistency, and applicability, the scheme was pilot tested on 20 abstracts by UB and JDL.

The overall level of PICO specification was calculated as a cumulative PICO score (minimum 0 and maximum 12), whereby a high level of specification for any PICO element was attributed a score of 3, medium=2, low=1, and not specified=0. Since 2 P-elements (P1 problem and P2 population) were considered, the average of both scores was calculated to avoid overweighting of the P-element.

#### Quantitative Descriptive and Correlation Analysis

We performed univariate analysis on the variables for study characteristics, the classification of PICO elements, and the classification of the likelihood for conclusive evidence, creating descriptive statistics, including frequency tables. Where available, metric variables (eg, cumulative PICO scores and publication year) were used in the analysis and complemented by group comparisons of their ordinal counterparts (eg, for search periods, databases, and included studies). Nominal variables (eg, meta-analysis and RCT focus) were handled appropriately using group comparisons.

Associations between PICO specification and the likelihood of conclusive evidence were explored by bivariate analysis resulting in cross-tabulations and box plots. To test potential correlations statistically, we used Spearman rank correlation coefficient (ρ) due to the ordinal nature of key variables. *P* values were reported for completeness, but following Andrade [10], interpretation emphasized effect sizes and directional trends over strict significance thresholds. Correlation strength was categorized based on the conventions described by Kuckartz et al [11].

## Results

### PRISMA Flowchart

Out of 21,161 initial records, 2528 studies remained after screening. From these, a random sample of 250 was drawn for detailed analysis.

**Figure 1.**
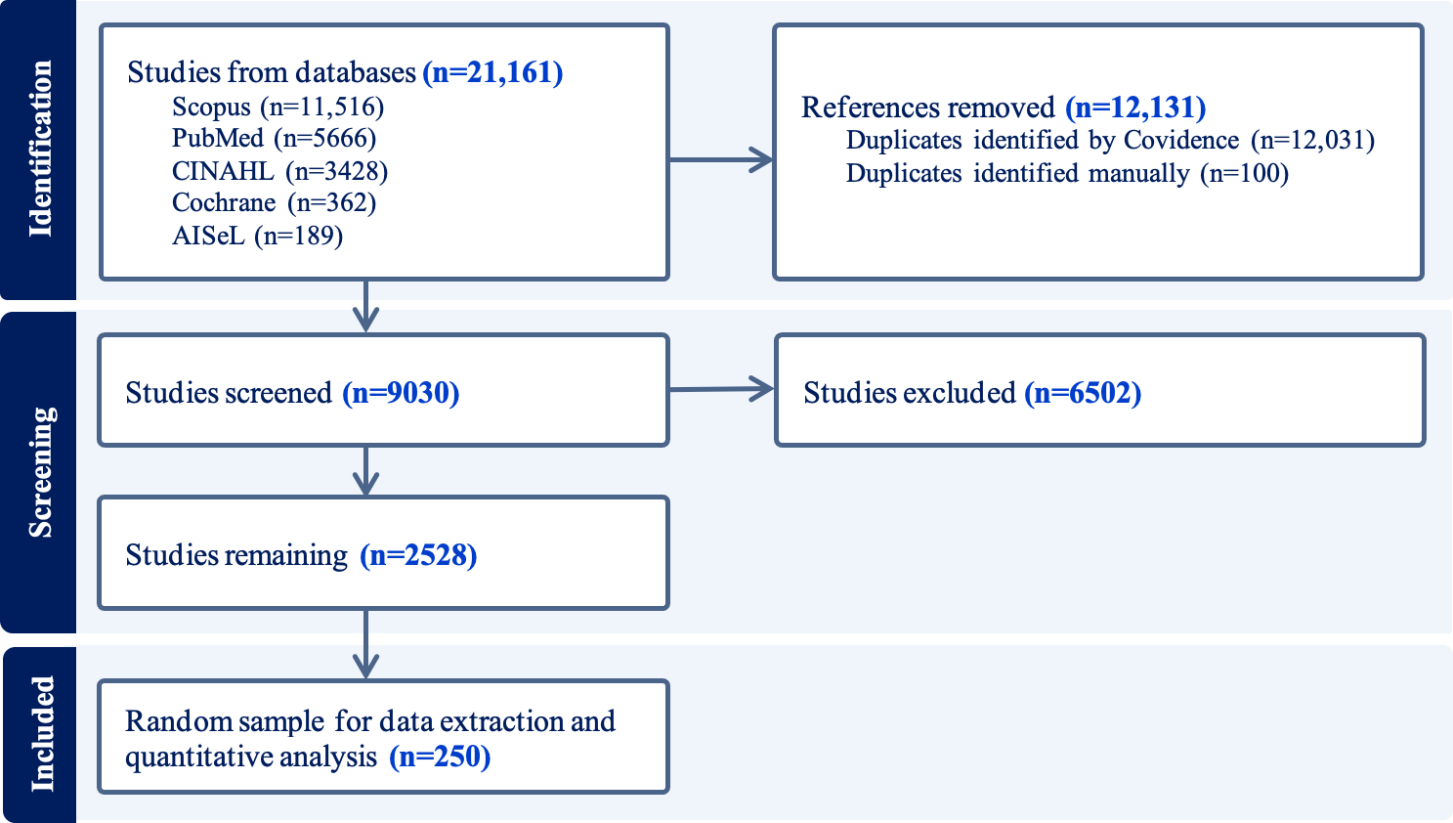
PRISMA (Preferred Reporting Items for Systematic Reviews and Meta-Analyses) flowchart.

Figure 1 shows the PRISMA flowchart.

### Study Characteristics in the Random Sample

We analyzed 9 study characteristics for the discussion in the context of their influence on evidence recognition and indications of possible methodological deficits.

Table 2 provides an overview, showing the number (n) of the 250 abstracts in which a study characteristic was reported together with the relative shares (%).

**Table 2.** Summary overview of study characteristics in the sample.

Study characteristics	Studies, n (%)
Study design	
Reported, of which:	250 (100)
Systematic review	146 (58.4)
Systematic review and meta-analysis	58 (23.2)
Systematic literature review	23 (9.2)
Meta-analysis	13 (5.2)
Systematic review of (systematic) reviews	6 (2.4)
Systematic review of systematic reviews and meta-analyses	4 (1.6)
Study types included	
Reported, of which:	170 (100)
RCTs^a^ only	62 (36.5)
RCTs among others	44 (25.9)
No RCTs	64 (37.6)
Not reported, n (% of 250)	80 (32)
Publication year	
Reported, of which:	250 (100)
2023	42 (16.8)
2022	59 (23.6)
2021	32 (12.8)
2020	21 (8.4)
2011‐2019	96 (38.4)
Search period in years	
Reported, of which:	159 (100)
1‐10	29 (18.2)
11‐20	32 (20.1)
>20 (eg, from inception)	98 (61.6)
Not reported, n (% of 250)	91 (36.4)
Number of databases searched	
Reported, of which:	202 (100)
1‐3	67 (33.2)
4‐6	102 (50.5)
7‐14	33 (16.3)
Not reported, n (% of 250)	48 (19.2)
Databases used (top 5)	
Reported, of which:	179 (100)
PubMed	110 (61.5)
Embase	107 (59.8)
MEDLINE (including Medline through PubMed)	102 (57)
CINAHL (EBSCO)	62 (34.6)
Web of Science	58 (32.4)
Not reported, n (% of 250)	80 (32)
Number of studies retrieved and screened	
Reported, of which:	82 (100)
1‐1000	28 (34.1)
1001‐2000	21 (25.6)
2001‐3000	10 (12.2)
3001‐4000	8 (9.8)
>4000	15 (18.3)
Not reported, n (% of 250)	168 (67.2)
Number of studies included	
Reported, of which:	235 (100)
1‐10	65 (27.7)
11‐20	75 (31.9)
21‐30	42 (17.9)
31‐40	23 (9.8)
>40	30 (12.8)
Not reported, n (% of 250)	15 (6)
Critical appraisal and RoB^b^ tools (top 5)	
Reported, of which:	76 (100)
Cochrane Collaboration Risk of Bias tools	27 (32.5)
GRADE^c^	10 (12)
Risk of bias assessment tools (unspecified)	7 (8.4)
Joanna Briggs Institute Critical Appraisal Checklist	6 (7.2)
AMSTAR^d^ or AMSTAR-2	5 (6)
Not reported, n (% of 250)	174 (69.6)

a RCT: randomized controlled trial.

b RoB: risk of bias.

c GRADE: Grading of Recommendations, Assessment, Development, and Evaluation.

d AMSTAR: A Measurement Tool to Assess Systematic Reviews.

- Study design: 30% (75/250) feature meta-analyses, 4% (10/250) were systematic reviews of (systematic) reviews.
- Study types: Overall, 32 study designs were mentioned in 170 abstracts; RCTs in 106 (62.4%), followed by controlled clinical trials (in 13, 7.6%), and pre-post studies (in 13, 7.6%), nonrandomized controlled trials in 12 (7.1%), and cohort studies in 10 (5.9%).
- Publication year: Over the last years, publications increased exponentially; 53.2% of studies (133/250) were published since 2021.
- Search period in years: Search periods were generally rather long, and “from inception” of the databases was often (27/159, 17%) specified.
- Number of databases searched: Just over half of the studies were based on the search in 4 to 6 databases, with a mean of 5 (SD 2) and a median of 4 (IQR 3-6). One-third of searches were performed in just 1-3 databases.
- Databases used: Overall, 65 databases were mentioned in 179 abstracts; 9 were mentioned in more than 10% of abstracts, 27 only once (1/179, 0.6%).
- Number of studies retrieved and screened: Researchers retrieved and screened between 49 and 42,946 studies with a median of 1491 (IQR 588‐3389) and a mean of 3016 (SD 5502).
- Number of studies included: Despite generally large volumes of studies screened, relatively small shares were included (median 1.2%, IQR 0.6‐3.1; mean 4.2%, SD 8%), ranging overall between 0.02% and 44.9%. The absolute numbers range from 1 to 236, with a median of 17 (IQR 10-30) and a mean of 25 (SD 29).
- Critical appraisal and risk of bias (RoB) tools: Overall, 29 tools were mentioned in 76 abstracts; 2 tools in 18 abstracts (23.7%). In this context, it is noteworthy that, if reported in the abstract, study or evidence quality was often described as low (19/42, 45%) and RoB as high (11/23, 48%).

### Level of Specification of PICO Elements

The level of specification of PICO elements was analyzed in the context of the quality of current research questions in the field of DHIs. Each PICO element plays a distinct role in framing the scope and analytical precision of a review. Their individual specifications were therefore examined separately.

**Figure 2.**
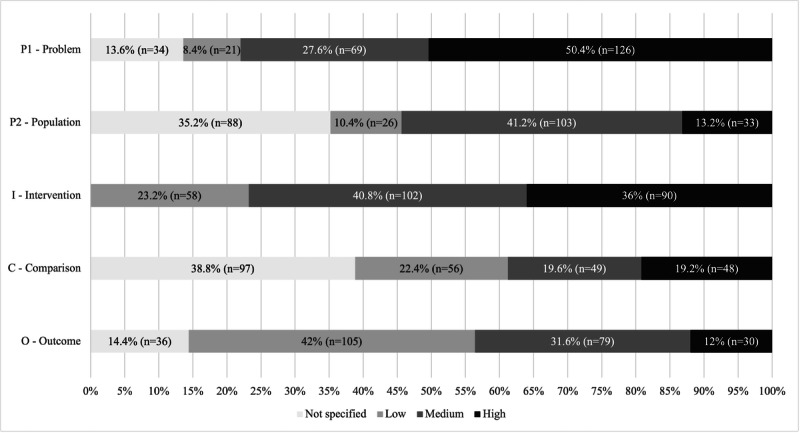
Level of specification of PICO (population, problem, or patient, intervention, comparison, and outcome) elements (% and n of sample, N=250).

Table 3 and Figure 2 display the level of specification of PICO elements according to the classification scheme (Table S1 in Multimedia Appendix 3) across the sample. A key finding is that no single PICO element was highly specified in more than half of the abstracts.

**Table 3.** Level of specification of PICO^a^ elements, criteria for classification.

PICO element, classification		Criteria for classification	Studies (N=250), n (%)
P1 (Problem: disorder or condition)			250 (100)
High		One medical problem or objective is clearly specified	126 (50.4)
Medium		A relatively homogenous group of medical problems or objectives	69 (27.6)
Low		Multiple highly heterogenous medical problems or objectives	21 (8.4)
Not specified		No specification whatsoever	34 (13.6)
P2 (Population: user group)			250 (100)
High		One, clearly specified, eg, by disorder AND additional characteristics	33 (13.2)
Medium		A relatively homogenous population or target group, eg, patients	103 (41.2)
Low		Multiple highly heterogenous populations or target groups	26 (10.4)
Not specified		No specification whatsoever	88 (35.2)
I (Intervention: technology)			250 (100)
High		One specific technology or aspect of homogenous technologies	90 (36)
Medium		A relatively homogenous group of technologies	102 (40.8)
Low		An unspecific range of highly heterogenous technologies	58 (23.2)
Not specified		No specification whatsoever	0 (0)
C (Comparison: comparable clinical practice or setting)			250 (100)
High		One care setting is clearly specified	48 (19.2)
Medium		A relatively homogenous group of care settings or explicit focus on intersectoral exchange	49 (19.6)
Low		An unspecific range of highly heterogenous care practices or settings, which may include no care, self-care, and multiple healthcare settings	56 (22.4)
Not specified		No specification whatsoever	97 (38.8)
O (Outcome)			250 (100)
High		One outcome is clearly specified with defined indicators	30 (12)
Medium		A relatively homogenous group of outcomes or a small number of specified and predefined but diverse outcomes of interest	79 (31.6)
Low		An unspecific range of heterogenous outcomes	105 (42)
Not specified		No focus on outcomes; outcomes are one of multiple objectives	36 (14.4)

a PICO: population, problem, or patient, intervention, comparison, and outcome.

Research focus on 1 medical problem (P1), such as the disease, condition, or clinical objective, was highly specified in 50.4% (126/250) of the abstracts. The intervention (I), referring to the technology used, was specified to some degree in all abstracts, with levels of specification distributed from low (58/250, 23.2%) to high (90/250, 36%). Outcomes (O) were relatively well-defined in 43.6% (109/250) of abstracts, whereas the remaining lacked a clear focus on predefined outcomes. Notably, 35.2% (88/250) of abstracts did not specify the population (P2), referring to the user group of the intervention, and 38.8% (97/250) lacked any specification for the comparison (C) element, referring to the health care setting applicable to the intervention. The population and comparison were highly specified in only 13.2% (33/250) and 19.2% (48/250) of abstracts, respectively.

### Level of PICO Specification and Likelihood of Conclusive Evidence

We hypothesized that low PICO specification in research objectives, methods, and questions is associated with low evidence recognition. Therefore, our study aimed to examine the relationship between PICO specification and the availability of conclusive evidence described in the abstracts.

To this end, criteria for the likelihood of conclusive evidence were defined based on literature and a qualitative review and thematic analysis of all results and conclusions reported in the abstracts (Table S2 in Multimedia Appendix 3).

Table 4 provides a summary of these criteria and the results of their application to the sample.

**Table 4.** Likelihood of conclusive evidence, criteria for the classification.

Likelihood of conclusive evidence, classification	Criteria for classification	Studies (N=250), n (%)
High	Meta-Analysis AND multiple (>1) “significant” results reported	19 (7.6)
Medium	Meta-Analysis AND at least comparable results to usual care OR no meta-analysis, but at least one “significant” effect reported for >25% of relevant studies	72 (28.8)
Low	Neither meta-analysis nor any “significant” results reported. Evidence is mainly or only reported qualitatively. If any quantitative results are reported, none is described as “significant.”	135 (54)
Inconclusive	Evidence is not reported at all OR without any directional conclusion OR although studies report positive evidence, also quantitatively, there are equally contradictive effects reported.	24 (9.6)

**Figure 3.**
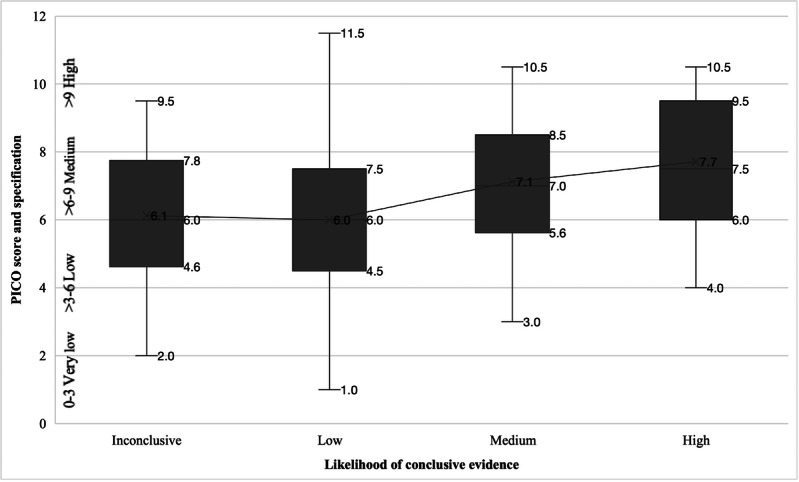
Distribution of the cumulative PICO score by likelihood of conclusive evidence classification. PICO: population, problem, or patient, intervention, comparison, and outcome.

To assess whether methodological focus correlates with result strength (our second research question), we examined the relationship between the overall level of PICO specification, based on a cumulative PICO score (minimum=0, maximum=12; 0‐3=very low, >3‐6=low, >6‐9=medium, and >9=high), and the likelihood of conclusive evidence being reported. Table 5 and Figures3  4 show the results.

**Table 5.** Correlation between overall PICO^a^ specification and likelihood of conclusive evidence (n [%] of sample, N=250).

Overall PICO specification	Likelihood of conclusive evidence				
	High	Medium	Low	Inconclusive	Total
High	6 (31.6)	11 (15.3)	10 (7.4)	3 (12.5)	30 (12)
Medium	8 (42.1)	38 (52.8)	48 (35.6)	7 (29.2)	101 (40.4)
Low	5 (26.3)	22 (30.6)	64 (47.4)	12 (50)	103 (41.2)
Very low	0 (0)	1 (1.4)	13 (9.6)	2 (8.3)	16 (6.4)
Total	19 (100)	72 (100)	135 (100)	24 (100)	250 (100)

a PICO: population, problem, or patient, intervention, comparison, and outcome.

**Figure 4.**
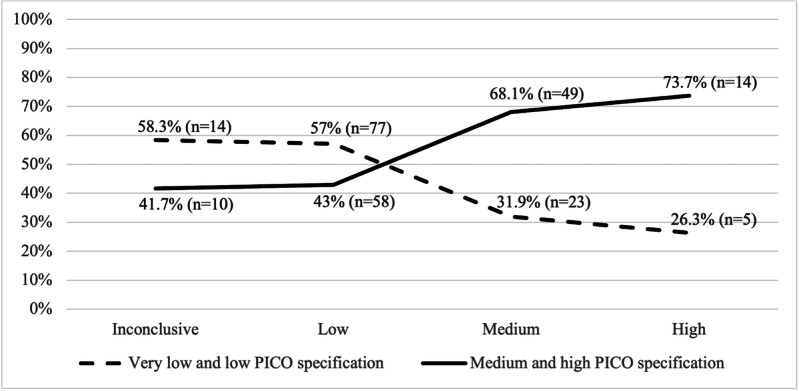
Combined PICO specification shares by likelihood of conclusive evidence classification, % (n) of abstracts in each evidence class. PICO: population, problem, or patient, intervention, comparison, and outcome.

Among the 30 abstracts (30/250, 12%) with a high PICO score, only 4 specified all 4, 20 specified 3, and 6 specified only 2 PICO elements highly. Of those 101 with a medium PICO score, only 6 specified 3, and 46 specified 2 PICO elements highly. The 103 abstracts with a low PICO score included only 8 with 2 PICO elements and 44 with 1 PICO element being highly specified.

This means that very few systematic reviews (4/250; 1.6%) have a clear focus on 1 problem or population, 1 intervention, 1 comparative setting, and 1 outcome. On the other hand, 47.6% (119/250) demonstrate a low or very low level of PICO specification, indicating lack of research focus and a very broad scope.

Only 6 abstracts in the sample reported highly conclusive positive evidence on highly specified PICO-based research questions (6/19, 31.6% of abstracts indicating highly conclusive evidence; 6/250, 2.4% of the entire sample).

Figure 3 and Figure 4 visualize the increasing likelihood of conclusive evidence with rising PICO specification. In Figure 3, PICO specification is represented by the PICO score.

Figure 4 illustrates the distribution of the combined shares for very low and low, and medium and high PICO specification within each evidence class, highlighting that methodological clarity alone does not guarantee robust findings.

While the combined medium and high PICO specification is associated with more robust findings, about two-fifths of the abstracts that remained inconclusive or yielded a low likeliness of conclusive evidence did so regardless of the medium or high level of PICO specification.

Correlation analysis confirmed a weak to medium positive relationship with statistical significance (ρ=0.26; *P*<.001) between overall PICO specification (ie, PICO score) and conclusive evidence. Higher specification of the outcome of interest (PICO element O; ρ=0.27; *P*<.001) and of the problem (PICO element P1; ρ=0.17; *P*=.008) were most associated with conclusive evidence being reported. In contrast, specification of the PICO elements population (P2), intervention (I), and comparison (C) showed no or only weak relationships with conclusive evidence.

### Methodological Characteristics and Likelihood of Conclusive Evidence

To illustrate the implications for future review practice, we have aligned the key insights of this study with the typical phases of a systematic review process, adapted from the core methods of conducting a Cochrane review [12,13].

**Figure 5.**
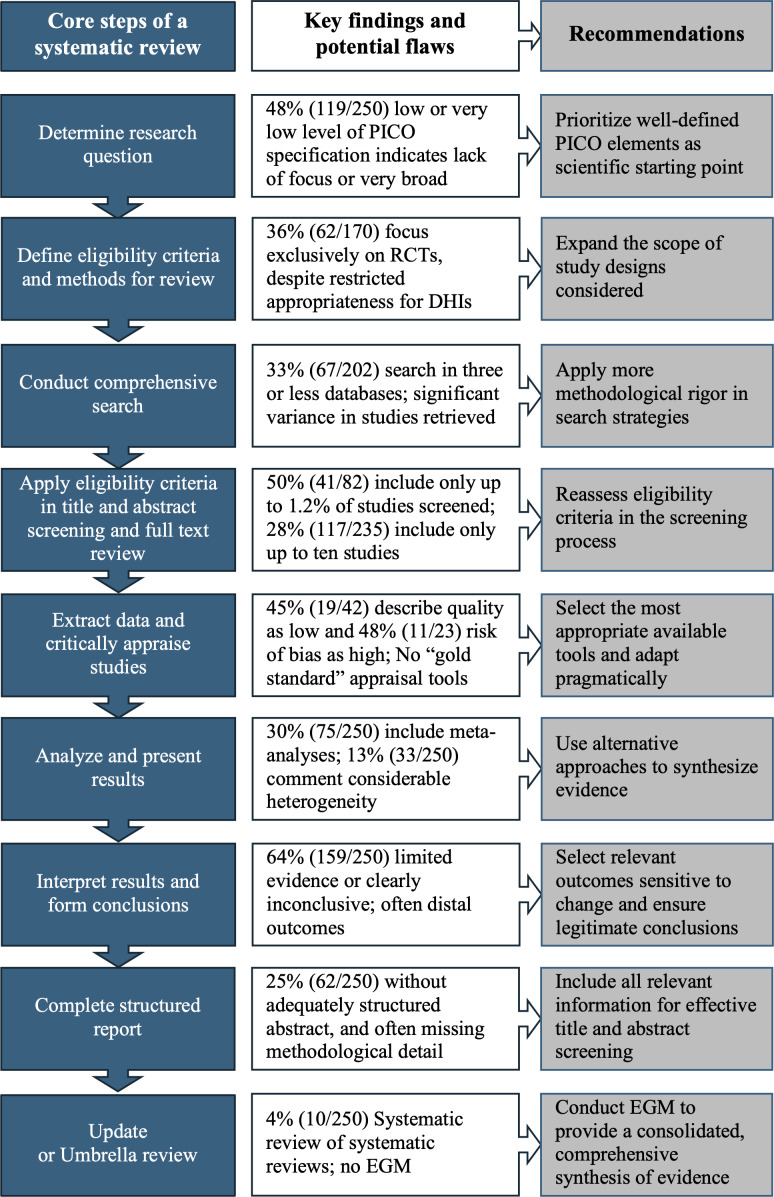
Overview of key findings and potential flaws, and recommendations along the steps of a systematic review. DHI: digital health intervention; EGM: evidence and gap mapping; PICO: population, problem, or patient, intervention, comparison, and outcome; RCT: randomized controlled trial.

Figure 5 highlights where critical flaws most frequently occur and also offers targeted suggestions to strengthen methodological coherence and improve evidence recognition, particularly in the context of complex interventions, such as DHIs.

In the interpretation of the above data, it should be noted that sample sizes (n) on which percentages are based vary. Refer to Tables2, 4,  5 and Multimedia Appendix 4 for details.

Research question: Our findings indicate inadequate application of PICO in formulating research questions, with 47.6% (119/250) of abstracts displaying low or very low PICO specification, and 63.6% (159/250) featuring poor or inconclusive evidence.

Although statistically significant, the correlation between PICO specification and conclusive evidence remains modest (ρ=0.26; *P*<.001). Outcome and problem specification showed the strongest associations with conclusive evidence, indicating their central role in the methodological quality of systematic reviews. Despite this apparent relationship, more than 40% of abstracts with poor (58/135) or inconclusive (10/24) evidence exhibit medium to high PICO specification.

Eligibility criteria: Currently, 36.5% (62/170) of systematic reviews rely exclusively on RCTs despite the availability of several alternatives, such as pre-post studies, nonrandomized controlled trials, cohort studies, time series, and case studies, that may also be appropriate, suggesting a need to expand the scope of study designs considered.

Search strategy: Despite more than 65 databases being available, 33.2% (67/202) of authors only used 3 or fewer databases. The number of studies retrieved and screened varies greatly, indicating significant variation in search strategy quality, particularly search periods and the formulation of search terms, operators, and search strings. Interestingly, no apparent relationship exists between PICO specification levels in the abstracts and search results. In total, 6 of the 10 lowest search results with less than 150 identified studies show poor PICO specification, while 6 of the 10 highest search results are associated with a high level of PICO specification.

Screening criteria: Despite generally large numbers of studies being identified and screened, only a small percentage (median 1.2%, IQR 0.6%‐3.1%; mean 4.2%, SD 8%) and absolute number (median 17; mean 25) is finally included, with 27.7% (65/235) of systematic reviews including 10 or less studies. In this context, it is noteworthy that 24.8% (62/250) of systematic review abstracts are not structured along background, methods, results, and conclusions, with missing details having a potential impact on the screening decision.

Critical appraisal tools: There is a host of tools available for different study designs (eg, systematic reviews, mixed methods, and primary research), research focus (eg, care practice, diagnostic accuracy, and health economics), and appraisal focus (eg, RoB and quality), of which 29 were identified in the sample. The choice of appraisal tool and the extreme variability in their criteria can significantly influence the exclusion of relevant studies, especially due to harsh appraisal ratings. Even for included studies, it is noteworthy that, if reported in the abstract, study or evidence quality is often described as low (19/42, 45%) and RoB as high (11/23, 48%). On the other hand, Blum et al [14] found no association with study quality for their results.

Evidence synthesis: Meta-analysis, the most desirable output of a systematic review to derive conclusive evidence, is featured in only 30% (75/250) of studies. Moreover, 13.2% (33/250) of abstracts include explicit comments on considerable heterogeneity or variability of study designs, populations, interventions, and outcomes, often stating that this prevented meta-analysis [15-25]. Alternative synthesis methods, such as thematic analysis [26] or statistical result aggregation [27,28], are underused. Higher-level syntheses, such as umbrella reviews of systematic reviews, are still rare (10/250, 4%).

Conclusion validity: In total, 54% (135/250) of systematic review abstracts reported neither meta-analysis nor any significant results; 9.6% (24/250) reported no evidence at all or no directional conclusion or equally contradictive effects. Although our analysis did not delve deeply into outcome effects, we stress the importance of selecting outcomes sensitive to change, as noted by Shen et al [29].

## Discussion

### Summary of Main Findings

This meta-research study aimed to identify key methodological factors that limit conclusive evidence recognition in systematic reviews on DHIs. Our findings show that the overall level of PICO specification in the abstracts was low or very low in nearly half of the sample, and that two-thirds of the reviews yielded inconclusive or weak evidence. Although higher levels of PICO specification, particularly regarding outcomes and clinical problems, were moderately associated with the likelihood of conclusive findings, these factors alone were not sufficient to guarantee evidence clarity.

This observation supports our initial assumption that PICO-structured research questions provide an important methodological foundation. However, it also reveals the limitations of assuming a linear relationship between methodological structure and evidentiary strength. Indeed, about two-fifths of reviews with low or inconclusive evidence had medium to high PICO specification. This suggests that even clearly defined research questions may fail to translate into robust evidence if other aspects of the review process are flawed.

Among these aspects, we identified recurring issues in search strategy design (eg, limited database use and vague search terms), restrictive eligibility criteria (eg, exclusive reliance on RCTs), and inconsistent use of quality appraisal tools. These elements interact with question formulation and may explain why even well-specified PICO frameworks can fail to yield strong evidence. Notably, substantial heterogeneity in study designs and outcomes further impedes evidence synthesis, particularly when meta-analyses are not feasible or misapplied.

### Detailed Discussion

Our findings underscore the need for a more integrated methodological approach. Rather than focusing solely on formal structures, review authors should align research questions, search strategies, eligibility and screening criteria, critical appraisal, evidence synthesis methods, and conclusions to the specific characteristics of digital health technologies. This is particularly relevant for DHIs, which often involve complex, context-sensitive mechanisms of action that challenge conventional evaluation models.

Research question: While high PICO specification does not guarantee conclusive results, a well-defined scientific starting point is critical [30]. In formulating the research question, both too specific and too broad specification of PICO elements appear more likely to lead to inconclusive results. We conclude, at a medium level of specification, that is, clearly defined and differentiated categories, in at least 2 or 3 PICO elements, would be optimal for many systematic reviews.

Eligibility criteria: Broad inclusion criteria, represented by low PICO specification, relate to substantial heterogeneity [30]. Conversely, narrow inclusion criteria, such as focusing solely on RCTs, also add challenges. Despite RCTs being the gold standard for intervention evaluations [31], they might not be fully appropriate in evaluating DHIs, due to long time frames, high costs, rigid protocols, and DHI specificities, such as individual tailoring and interaction effects that RCTs can insufficiently address [32-34].

Search strategy: A comprehensive and sensitive search strategy in multiple databases is recommended [30]. Our findings highlight the necessity for more methodological rigor in comprehensive search strategies to mitigate the inconsistent retrieval of relevant studies. This concerns particularly searches across multiple databases and search terms and strings that are more aligned with the research questions. Exponential growth in the number of publications and increasing quality of studies over time leads us to suggest restricting search periods for future systematic reviews to a maximum of 10 years.

Screening criteria: Our findings suggest that screening criteria might not effectively capture all relevant research to ensure comprehensive evidence synthesis, necessitating a reassessment in the screening process. Given the limitations observed with broad and narrow eligibility criteria, more consideration of inclusion and exclusion criteria, especially a focus on RCTs, seems to be required to balance comprehensiveness and specificity.

While the abstract structure and length may reflect specific journal guidelines, evolving reporting guidance should ensure that all standard structural components of a systematic review abstract (ie, background, methods, results, and conclusions) are included.

Critical appraisal tools: With 121 different tools published, “there is no ‘gold standard’ for any study design, nor is there any widely accepted generic tool that can be applied equally well across study types” [35]. This issue emphasizes the importance of selecting the most appropriate available tools and the need for more standardized, pragmatic tools that align with the objectives of systematic reviews on DHI effects.

The appraisal process may demand an assessment or validation and consolidation or adaptation of available tools to ensure it is capturing pertinent research without unnecessarily excluding valuable studies due to overly stringent or irrelevant criteria of appraisal tools.

Evidence synthesis: Incorporating alternative synthesis methods beyond meta-analysis is recommended. Meta-analysis is often challenged by substantial heterogeneity and variability in research questions, study designs, interventions, and outcomes, and incomparable data from the primary research studies. Recognizing the challenges of meta-analyses, systematic reviews should more often explore thematic analyses and other quantitative aggregations to ensure a comprehensive evidence base. Purely narrative approaches or summary tables of primary studies lack the quantitative aggregation needed for conclusive reviews.

Conclusion validity: Researchers should strive for outcome measures that are sensitive to change and aim to capture both relevant proximal and distal intervention effects to ensure meaningful and legitimate conclusions. In interpreting findings, conclusions that suggest limited evidence of benefits due to outcomes comparable with standard or conventional care may neglect the significance of parity when additional benefits, such as cost reduction, improved timeliness, and enhanced access to care, are considered. This is particularly pertinent to telemedicine, telerehabilitation, and telehealth.

### Limitations

This study has several limitations that should be considered when interpreting the findings.

First, all data were extracted exclusively from abstracts. We acknowledge that abstracts may not fully capture the methodological details of the full texts; our analysis, therefore, reflects reported, not necessarily executed, methodological rigor. However, abstracts are the primary entry point for study screening and selection, making them critical for evidence recognition.

Second, the analysis was descriptive and exploratory in nature. No causal claims can be made, and the moderate correlations observed should be interpreted as indicative rather than predictive.

Third, data extraction and analysis were based on a random sample rather than a full systematic review of all eligible studies. Thus, while the sample was randomly drawn and diverse in terms of publication outlets and topics, it may not fully represent the entire body of systematic reviews on DHIs.

Finally, although a structured coding scheme was applied, some degree of subjective judgment was inevitable in the classification of PICO elements and evidence conclusiveness.

Future studies should complement abstract-level analyses with full-text reviews, interrater reliability testing, and more refined models to account for complex interactions between review components.

### Conclusions

This sample-based meta-research study provides empirical insight into why systematic reviews on DHIs often fail to yield conclusive evidence.

The findings underscore the crucial role of optimally specifying the PICO elements of interest in DHI research, revealing that inadequate PICO specification correlates somewhat with suboptimal evidence recognition.

However, while clearer specification—especially of outcomes and clinical problems—was moderately associated with stronger evidence, this alone did not guarantee evidentiary clarity.

Our findings suggest that methodological coherence across all review stages is a necessary condition to ensure conclusions and evidence-informed decisions in the digitalization of healthcare are both valid and meaningful.

In summary, beyond question formulation, issues in search strategy, eligibility criteria, and synthesis design also play a critical role in shaping review outcomes. To support the underlying processes, we propose a structured, PICO-based categorization framework that is aligned with this research, builds on well-established categories, and provides clear and differentiated definitions and associated terms. Such a framework could inform the research question, and the search strategy and screening process by providing terms for search strings and inclusion or exclusion criteria. Our proposed framework could ultimately enable comprehensive and continuous evidence and gap mapping for DHIs, especially if the increasing power of artificial intelligence tools is leveraged in the research process. Thus, it may help to improve the quality and transparency of systematic reviews—especially in the complex and dynamic field of digital health.

## Supplementary material

10.2196/78210 Multimedia Appendix 1 Search strings by database.

10.2196/78210 Multimedia Appendix 2 Inclusion and exclusion criteria.

10.2196/78210 Multimedia Appendix 3 Classification criteria.

10.2196/78210 Multimedia Appendix 4 Sample, charting, and analyses.

10.2196/78210 Checklist 1 PRISMA-ScR checklist.

## Abbreviations

DHI: digital health intervention
PICO: population, problem, or patient, intervention, comparison, and outcome
PRISMA-ScR: Preferred Reporting Items for Systematic Reviews and Meta-Analyses extension for Scoping Reviews
RCT: randomized controlled trial
RoB: risk of bias

## References

[1] Aagja J, Shome S, Chandra A (2023). A bibliometric analysis of digital health & mobile health related global research publications. Hosp Top.

[2] Babić A, Poklepović Peričić T, Pieper D, Puljak L (2022). When is the evidence conclusive? Analysis of systematic reviews for which Cochrane declared that conclusions will not change with further studies. Res Synth Methods.

[3] Kang T, Zou S, Weng C (2019). Pretraining to recognize PICO elements from randomized controlled trial literature. Stud Health Technol Inform.

[4] Richardson WS, Wilson MC, Nishikawa J, Hayward RS (1995). The well-built clinical question: a key to evidence-based decisions. ACP J Club.

[5] Cañón M, Buitrago-Gómez Q (2018). The research question in clinical practice: a guideline for its formulation. Revista Colombiana de Psiquiatría (English ed).

[6] Hosseini MS, Jahanshahlou F, Akbarzadeh MA, Zarei M, Vaez-Gharamaleki Y (2024). Formulating research questions for evidence-based studies. J Med Surg Public Health.

[7] Huang X, Lin J, Demner-Fushman D (2006). Evaluation of PICO as a knowledge representation for clinical questions. AMIA Annu Symp Proc.

[8] Schardt C, Adams MB, Owens T, Keitz S, Fontelo P (2007). Utilization of the PICO framework to improve searching PubMed for clinical questions. BMC Med Inform Decis Mak.

[9] Tricco AC, Lillie E, Zarin W (2018). PRISMA Extension for Scoping Reviews (PRISMA-ScR): checklist and explanation. Ann Intern Med.

[10] Andrade C (2019). The *P* value and statistical significance: misunderstandings, explanations, challenges, and alternatives. Indian J Psychol Med.

[11] Kuckartz U, Rädiker S, Ebert T, Schehl J (2013). Statistik: Eine Verständliche Einführung 2, Überarbeitete Auflage.

[12] (2011). Session three: a “snapshot” of the steps of conducting a Cochrane Review (part 1). YouTube.

[13] Higgins JPT, Thomas J, Chandler J (2024). Part 2: Core Methods.

[14] Blum D, Raj SX, Oberholzer R (2015). Computer-based clinical decision support systems and patient-reported outcomes: a systematic review. Patient.

[15] Buford A, Ashworth HC, Ezzeddine FL (2022). Systematic review of electronic health records to manage chronic conditions among displaced populations. BMJ Open.

[16] Rostam Niakan Kalhori S, Rahmani Katigari M, Talebi Azadboni T, Pahlevanynejad S, Hosseini Eshpala R (2023). The effect of m-health applications on self-care improvement in older adults: a systematic review. Inform Health Soc Care.

[17] Smith C, Gold J, Ngo TD, Sumpter C, Free C (2015). Mobile phone-based interventions for improving contraception use. Cochrane Database Syst Rev.

[18] Jones OT, Calanzani N, Saji S (2021). Artificial intelligence techniques that may be applied to primary care data to facilitate earlier diagnosis of cancer: systematic review. J Med Internet Res.

[19] Daryabeygi-Khotbehsara R, Shariful Islam SM, Dunstan D, McVicar J, Abdelrazek M, Maddison R (2021). Smartphone-based interventions to reduce sedentary behavior and promote physical activity using integrated dynamic models: systematic review. J Med Internet Res.

[20] Aluga D, Nnyanzi LA, King N, Okolie EA, Raby P (2021). Effect of electronic prescribing compared to paper-based (handwritten) prescribing on primary medication adherence in an outpatient setting: a systematic review. Appl Clin Inform.

[21] Veldhuis LI, Woittiez NJC, Nanayakkara PWB, Ludikhuize J (2022). Artificial intelligence for the prediction of in-hospital clinical deterioration: a systematic review. Crit Care Explor.

[22] Akazawa M, Hashimoto K (2021). Artificial intelligence in gynecologic cancers: current status and future challenges - a systematic review. Artif Intell Med.

[23] Sondaal SFV, Browne JL, Amoakoh-Coleman M, Li D (2016). Assessing the effect of mHealth interventions in improving maternal and neonatal care in low- and middle-income countries: a systematic review. PLOS ONE.

[24] McAlpine H, Joubert L, Martin-Sanchez F, Merolli M, Drummond KJ (2015). A systematic review of types and efficacy of online interventions for cancer patients. Patient Educ Couns.

[25] Paalimäki-Paakki K, Virtanen M, Henner A, Nieminen MT, Kääriäinen M (2022). Effectiveness of digital counseling environments on anxiety, depression, and adherence to treatment among patients who are chronically ill: systematic review. J Med Internet Res.

[26] Bul KCM, Bannon C, Krishnan N, Dunlop A, Szczepura A (2023). Can eHealth applications improve renal transplant outcomes for adolescents and young adults? A systematic review. Transplant Rev (Orlando).

[27] Edison MA, Connor MJ, Miah S (2020). Understanding virtual urology clinics: a systematic review. BJU Int.

[28] Mokaya M, Kyallo F, Vangoitsenhoven R, Matthys C (2022). Clinical and patient-centered implementation outcomes of mHealth interventions for type 2 diabetes in low-and-middle income countries: a systematic review. Int J Behav Nutr Phys Act.

[29] Shen H, van der Kleij R, van der Boog PJM, Chang X, Chavannes NH (2019). Electronic health self-management interventions for patients with chronic kidney disease: systematic review of quantitative and qualitative evidence. J Med Internet Res.

[30] Greco T, Zangrillo A, Biondi-Zoccai G, Landoni G (2013). Meta-analysis: pitfalls and hints. Heart Lung Vessel.

[31] Piantadosi S (2005). Clinical Trials: A Methodologic Perspective.

[32] Hrynyschyn R, Prediger C, Stock C, Helmer SM (2022). Evaluation methods applied to digital health interventions: what is being used beyond randomised controlled trials?-A scoping review. Int J Environ Res Public Health.

[33] Guo C, Ashrafian H, Ghafur S, Fontana G, Gardner C, Prime M (2020). Challenges for the evaluation of digital health solutions-a call for innovative evidence generation approaches. NPJ Digit Med.

[34] Ahuja AS (2019). Should RCT’s be used as the gold standard for evidence based medicine?. Integr Med Res.

[35] Katrak P, Bialocerkowski AE, Massy-Westropp N, Kumar VS, Grimmer KA (2004). A systematic review of the content of critical appraisal tools. BMC Med Res Methodol.

