# Supplementary material for: Evaluating Methodological Coherence and Evidence Recognition in Digital Health Systematic Reviews: Sample-based Meta-research Study
Source: Online J Public Health Inform. 2026 Apr 16;18:e78210. doi: 10.2196/78210 (PMC13086262; doi:10.2196/78210)
Supplement: Multimedia Appendix 1 [file ojphi-v18-e78210-s001.docx]

# Multimedia Appendix 1 Search strings by database

## MEDLINE via PubMed

1. decision support systems, clinical [mh]
2. medical records systems, computerized [mh]
3. reminder systems [mh]
4. health records, personal [mh]
5. telemedicine [mh]
6. hospital information systems [mh]
7. nursing informatics [mh]
8. health information interoperability [mh]
9. consumer health informatics [mh]
10. artificial intelligence [mh:noexp]
11. big data [mh]
12. radiology information systems[mh]
13. OR/1-12
14. "health information technolog*"[tiab]
15. "clinical information system*"[tiab]
16. "clinical decision support"[tiab]
17. "electronic health record*"[tiab]
18. "electronic medical record*"[tiab]
19. "health smart card*"[tiab]
20. "health information exchange"[tiab]
21. "reminder system*"[tiab]
22. "personal health record*"[tiab]
23. "patient portal*"[tiab]
24. "telemedicine"[tiab]
25. "mhealth"[tiab]
26. "ehealth"[tiab]
27. "telehealth"[tiab]
28. "mobile health"[tiab]
29. "hospital information system*"[tiab]
30. "computerized provider order entry"[tiab]
31. "closed loop medic*""[tiab]
32. "nursing informatic*"[tiab]
33. "nursing information system*"[tiab]
34. "interoperab*"[tiab]
35. "consumer health informatic*"[tiab]
36. "cdss"[tiab]
37. "cpoe"[tiab]
38. "ehr"[tiab]
39. "emr"[tiab]
40. "artificial intelligence"[tiab]
41. "blockchain*"[tiab]
42. "distributed ledger*"[tiab]
43. "e-health"[tiab]
44. "radiology information system*"[tiab]
45. "picture archiving and communication system*"[tiab]
46. "pacs"[tiab]
47. OR/14-46
48. 13 OR 47
49. quality improvement [mh:noexp]
50. meaningful use [mh]
51. quality of health care [mh:noexp]
52. outcome and process assessment, health care [mh]
53. efficiency [mh]
54. costs and cost analysis [mh]
55. patient safety [mh]
56. medical errors [mh]
57. medication errors [mh]
58. empowerment [mh]
59. patient acceptance of health care [mh]
60. digital divide [mh]
61. learning health system [mh]
62. health services accessibility [mh]
63. health equity [mh]
64. OR/49-63
65. "quality improvement*"[tiab]
66. "meaningful use"[tiab]
67. "quality of health care”[tiab]
68. "quality of healthcare”[tiab]
69. "health care quality”[tiab]
70. "healthcare quality”[tiab]
71. "quality of care”[tiab]
72. "care quality”[tiab]
73. "patient outcome*”[tiab]
74. "treatment outcome*”[tiab]
75. "patient relevant outcome*”[tiab]
76. "effectiveness”[tiab]
77. "efficiency”[tiab]
78. "productivity”[tiab]
79. "cost-effectiveness”[tiab]
80. "cost effectiveness”[tiab]
81. "cost benefit*”[tiab]
82. "cost-benefit*”[tiab]
83. "cost saving*”[tiab]
84. "patient safety”[tiab]
85. "medical error*”[tiab]
86. "medication error*”[tiab]
87. "prescription error*”[tiab]
88. "patient acceptance of health care”[tiab]
89. "health care utilization”[tiab]
90. "healthcare utilization”[tiab]
91. "patient compliance”[tiab]
92. "medication adherence”[tiab]
93. "drug adherence”[tiab]
94. "medication compliance”[tiab]
95. "patient participation”[tiab]
96. "patient engagement”[tiab]
97. "digital divide”[tiab]
98. "health equity”[tiab]
99. "learning health system*”[tiab]
100. "access to health”[tiab]
101. "accessibility”[tiab]
102. "prescribing error*”[tiab]
103. "unintended consequence*”[tiab]
104. "adverse effect*”[tiab]
105. "outcome*”[tiab]
106. OR/64-104
107. 64 OR 106
108. y_10[Filter]
109. fha[Filter]
110. meta-analysis[Filter]
111. systematicreview[Filter]
112. 110 OR 111
113. english[Filter]
114. german[Filter]
115. 113 OR 114
116. 108 AND 109 AND 112 AND 115
117. 48 AND 107
118. 116 AND 117

## Cochrane Library

1. [mh "decision support systems, clinical"]
2. [mh "medical records systems, computerized"]
3. [mh "reminder systems"]
4. [mh "health records, personal"]
5. [mh "telemedicine"]
6. [mh "hospital information systems"]
7. [mh "nursing informatics"]
8. [mh "health information interoperability"]
9. [mh "consumer health informatics"]
10. [mh ^"artificial intelligence"]
11. [mh "Big Data"]
12. [mh "Radiology Information Systems"]
13. {or #1-#12}
14. (health information technolog*):ti,ab,kw
15. ("clinical information system*"):ti,ab,kw
16. ("clinical decision support"):ti,ab,kw
17. ("electronic health record*"):ti,ab,kw
18. ("electronic medical record*"):ti,ab,kw
19. ("health smart card*"):ti,ab,kw
20. ("health information exchange"):ti,ab,kw
21. ("reminder system*"):ti,ab,kw
22. ("personal health record*"):ti,ab,kw
23. ("patient portal*"):ti,ab,kw
24. (telemedicine):ti,ab,kw
25. (mhealth):ti,ab,kw
26. (ehealth):ti,ab,kw
27. (telehealth):ti,ab,kw
28. ("mobile health"):ti,ab,kw
29. ("hospital information system*"):ti,ab,kw
30. ("computerized provider order entry"):ti,ab,kw
31. ("closed loop medic*"):ti,ab,kw
32. ("nursing informatic*"):ti,ab,kw
33. ("nursing information system*"):ti,ab,kw
34. (interoperab*):ti,ab,kw
35. ("consumer health informatic*"):ti,ab,kw
36. (CDSS):ti,ab,kw
37. (CPOE):ti,ab,kw
38. (EHR):ti,ab,kw
39. (EMR):ti,ab,kw
40. ("artificial intelligence"):ti,ab,kw
41. (blockchain*):ti,ab,kw
42. ("distributed ledger*"):ti,ab,kw
43. (e-health):ti,ab,kw
44. ("radiology information system*"):ti,ab,kw
45. ("picture archiving and communication system*"):ti,ab,kw
46. (PACS):ti,ab,kw
47. {or #14-#46}
48. #13 or #47
49. [mh ^"quality improvement"]
50. [mh "meaningful use"]
51. [mh ^"quality of health care"]
52. [mh "outcome and process assessment, health care"]
53. [mh "efficiency"]
54. [mh "costs and cost analysis"]
55. [mh "patient safety"]
56. [mh "medical errors"]
57. [mh "medication errors"]
58. [mh "empowerment"]
59. [mh "patient acceptance of health care"]
60. [mh "digital divide"]
61. [mh "learning health system"]
62. [mh ^"Health Services Accessibility"]
63. [mh "Health Equity"]
64. {or #49-#63}
65. ("quality improvement*"):ti,ab,kw
66. ("meaningful use"):ti,ab,kw
67. ("quality of health care"):ti,ab,kw
68. ("quality of healthcare"):ti,ab,kw
69. ("health care quality"):ti,ab,kw
70. ("healthcare quality"):ti,ab,kw
71. ("quality of care"):ti,ab,kw
72. ("care quality"):ti,ab,kw
73. ("patient outcome*"):ti,ab,kw
74. ("treatment outcome*"):ti,ab,kw
75. ("patient relevant outcome*"):ti,ab,kw
76. (effectiveness):ti,ab,kw
77. (efficiency):ti,ab,kw
78. (productivity):ti,ab,kw
79. ("cost-effectiveness"):ti,ab,kw
80. ("cost effectiveness"):ti,ab,kw
81. ("cost benefit*"):ti,ab,kw
82. ("cost-benefit*"):ti,ab,kw
83. ("cost saving*"):ti,ab,kw
84. ("patient safety"):ti,ab,kw
85. ("medical error*"):ti,ab,kw
86. ("medication error*"):ti,ab,kw
87. ("prescription error*"):ti,ab,kw
88. ("patient acceptance of health care"):ti,ab,kw
89. ("health care utilization"):ti,ab,kw
90. ("healthcare utilization"):ti,ab,kw
91. ("patient compliance"):ti,ab,kw
92. ("medication adherence"):ti,ab,kw
93. ("drug adherence"):ti,ab,kw
94. ("medication compliance"):ti,ab,kw
95. ("patient participation"):ti,ab,kw
96. ("patient engagement"):ti,ab,kw
97. ("digital divide"):ti,ab,kw
98. ("health equity"):ti,ab,kw
99. ("learning health system*"):ti,ab,kw
100. ("access to health"):ti,ab,kw
101. ("accessibility"):ti,ab,kw
102. ("prescribing error*"):ti,ab,ke
103. ("unintended consequence*"):ti,ab,kw
104. ("adverse effect*"):ti,ab,kw
105. ("outcome*"):ti,ab,kw
106. {or #65-#106}
107. #64 or #107
108. #107 and #48 with Cochrane Library publication date Between Jan 2011 and Oct 2023, in Cochrane Reviews

## CINAHL

1. MH "Clinical InformationSystems+"
2. MH "Reminder Systems"
3. MH "telehealth+"
4. MH"nursing informatics"
5. MH "Artificial Intelligence"
6. MH "medicalinformatics"
7. MH"hospital informationsystems"
8. MH"Decision SupportSystems, Clinical"
9. MH"Robotics"
10. OR/1-9
11. TI AB "health information technolog*"
12. TI AB "clinical information system*"
13. TI AB "clinical decision support"
14. TI AB "electronic health record*"
15. TI AB "electronic medical record*"
16. TI AB "health smart card*"
17. TI AB "health information exchange"
18. TI AB "reminder system*"
19. TI AB "personal health record*"
20. TI AB "patient portal*"
21. TI AB "telemedicine"
22. TI AB "mhealth"
23. TI AB "ehealth"
24. TI AB "telehealth"
25. TI AB "mobile health"
26. TI AB "hospital information system*"
27. TI AB "computerized provider order entry"
28. TI AB "closed loop medic*""
29. TI AB "nursing informatic*"
30. TI AB "nursing information system*"
31. TI AB "interoperab*"
32. TI AB "consumer health informatic*"
33. TI AB "cdss"
34. TI AB "cpoe"
35. TI AB "ehr"
36. TI AB "emr"
37. TI AB "artificial intelligence"
38. TI AB "blockchain*"
39. TI AB "distributed ledger*"
40. TI AB "e-health"
41. TI AB "radiology information system*"
42. TI AB "picture archiving and communication system*"
43. TI AB "pacs"
44. OR/11-43
45. 10 OR 44
46. MH "quality improvement"
47. MH "meaningful use"
48. MH "quality of health care"
49. MH "productivity"
50. MH "costs and cost analysis+"
51. MH "patient safety+"
52. MH "adverse health care event+"
53. MH "empowerment"
54. MH "digital divide"
55. MH "learning health system"
56. MH "health services accessibility"
57. MH "clinical documentation improvement"
58. MH "outcomes (health care)+"
59. MH "quality of nursing"
60. MH "organizational efficiency"
61. MH "attitude of health personnel+"
62. OR/46-61
63. TI AB "quality improvement*"
64. TI AB "meaningful use"
65. TI AB "quality of health care”
66. TI AB "quality of healthcare”
67. TI AB "health care quality”
68. TI AB "healthcare quality”
69. TI AB "quality of care”
70. TI AB "care quality”
71. TI AB "patient outcome*”
72. TI AB "treatment outcome*”
73. TI AB "patient relevant outcome*”
74. TI AB "effectiveness”
75. TI AB "efficiency”
76. TI AB "productivity”
77. TI AB "cost-effectiveness”
78. TI AB "cost effectiveness”
79. TI AB "cost benefit*”
80. TI AB "cost-benefit*”
81. TI AB "cost saving*”
82. TI AB "patient safety”
83. TI AB "medical error*”
84. TI AB "medication error*”
85. TI AB "prescription error*”
86. TI AB "patient acceptance of health care”
87. TI AB "health care utilization”
88. TI AB "healthcare utilization”
89. TI AB "patient compliance”
90. TI AB "medication adherence”
91. TI AB "drug adherence”
92. TI AB "medication compliance”
93. TI AB "patient participation”
94. TI AB "patient engagement”
95. TI AB "digital divide”
96. TI AB "health equity”
97. TI AB "learning health system*”
98. TI AB "access to health”
99. TI AB "accessibility”
100. TI AB "prescribing error*”
101. TI AB "unintended consequence*”
102. TI AB "adverse effect*”
103. TI AB "outcome*”
104. OR/63-103
105. 62 OR 104
106. 45 AND 105 with Limiters AbstractAvailable; Published Date:20110101-20231031;Publication Type: MetaAnalysis, SystematicReview; Language:English, German

## Scopus

1. TITLE-ABS ("health information technolog*")
2. TITLE-ABS ("clinical information system*")
3. TITLE-ABS ("clinical decision support")
4. TITLE-ABS ("electronic health record*")
5. TITLE-ABS ("electronic medical record*")
6. TITLE-ABS ("health smart card*")
7. TITLE-ABS ("health information exchange")
8. TITLE-ABS ("reminder system*")
9. TITLE-ABS ("personal health record*")
10. TITLE-ABS ("patient portal*")
11. TITLE-ABS ("telemedicine")
12. TITLE-ABS ("mhealth")
13. TITLE-ABS ("ehealth")
14. TITLE-ABS ("telehealth")
15. TITLE-ABS ("mobile health")
16. TITLE-ABS ("hospital information system*")
17. TITLE-ABS ("computerized provider order entry")
18. TITLE-ABS ("closed loop medic*"")
19. TITLE-ABS ("nursing informatic*")
20. TITLE-ABS ("nursing information system*")
21. TITLE-ABS ("interoperab*")
22. TITLE-ABS ("consumer health informatic*")
23. TITLE-ABS ("cdss")
24. TITLE-ABS ("cpoe")
25. TITLE-ABS ("ehr")
26. TITLE-ABS ("emr")
27. TITLE-ABS ("artificial intelligence")
28. TITLE-ABS ("blockchain*")
29. TITLE-ABS ("distributed ledger*")
30. TITLE-ABS ("e-health")
31. TITLE-ABS ("radiology information system*")
32. TITLE-ABS ("picture archiving and communication system*")
33. TITLE-ABS ("pacs")
34. OR/1-33
35. TITLE-ABS ("quality improvement*")
36. TITLE-ABS-KEY ("meaningful use")
37. TITLE-ABS ("quality of health care”)
38. TITLE-ABS ("quality of healthcare”)
39. TITLE-ABS ("health care quality”)
40. TITLE-ABS ("healthcare quality”)
41. TITLE-ABS ("quality of care”)
42. TITLE-ABS ("care quality”)
43. TITLE-ABS ("patient outcome*”)
44. TITLE-ABS ("treatment outcome*”)
45. TITLE-ABS ("patient relevant outcome*”)
46. TITLE-ABS ("effectiveness”)
47. TITLE-ABS ("efficiency”)
48. TITLE-ABS ("productivity”)
49. TITLE-ABS ("cost-effectiveness”)
50. TITLE-ABS ("cost effectiveness”)
51. TITLE-ABS ("cost benefit*”)
52. TITLE-ABS ("cost-benefit*”)
53. TITLE-ABS ("cost saving*”)
54. TITLE-ABS ("patient safety”)
55. TITLE-ABS ("medical error*”)
56. TITLE-ABS ("medication error*”)
57. TITLE-ABS ("prescription error*”)
58. TITLE-ABS ("patient acceptance of health care”)
59. TITLE-ABS ("health care utilization”)
60. TITLE-ABS ("healthcare utilization”)
61. TITLE-ABS ("patient compliance”)
62. TITLE-ABS ("medication adherence”)
63. TITLE-ABS ("drug adherence”)
64. TITLE-ABS ("medication compliance”)
65. TITLE-ABS ("patient participation”)
66. TITLE-ABS ("patient engagement”)
67. TITLE-ABS ("digital divide”)
68. TITLE-ABS ("health equity”)
69. TITLE-ABS ("learning health system*”)
70. TITLE-ABS ("access to health”)
71. TITLE-ABS ("accessibility”)
72. TITLE-ABS ("prescribing error*”)
73. TITLE-ABS ("unintended consequence*”)
74. TITLE-ABS ("adverse effect*”)
75. TITLE-ABS ("outcome*”)
76. OR/35-75
77. 34 AND 76
78. 77 AND ( PUBYEAR > 2010 ) AND TITLE-ABS-KEY ( "systematic review" OR "meta-analysis" ) AND ( LIMIT-TO ( SRCTYPE , "j" ) OR LIMIT-TO ( SRCTYPE , "p" ) ) AND ( EXCLUDE ( PUBYEAR , 2022 ) ) AND ( LIMIT-TO ( LANGUAGE , "English" ) OR LIMIT-TO ( LANGUAGE , "German" ) ) AND ( EXCLUDE ( SUBJAREA , "AGRI" ) OR EXCLUDE ( SUBJAREA , "CENG" ) OR EXCLUDE ( SUBJAREA , "DENT" ) OR EXCLUDE ( SUBJAREA , "MATE" ) OR EXCLUDE ( SUBJAREA , "PHYS" ) OR EXCLUDE ( SUBJAREA , "ENER" ) OR EXCLUDE ( SUBJAREA , "VETE" ) OR EXCLUDE ( SUBJAREA , "CHEM" ) OR EXCLUDE ( SUBJAREA , "EART" ) ) AND ( EXCLUDE ( DOCTYPE , "no" ) OR EXCLUDE ( DOCTYPE , "ed" ) OR EXCLUDE ( DOCTYPE , "le" ) OR EXCLUDE ( DOCTYPE , "sh" ) OR EXCLUDE ( DOCTYPE , "cr" ) OR EXCLUDE ( DOCTYPE , "er" ) )

## AISeL

1. "health information technolog*"
2. "clinical information system*"
3. "clinical decision support"
4. "electronic health record*"
5. "electronic medical record*"
6. "health smart card*"
7. "health information exchange"
8. "reminder system*"
9. "personal health record*"
10. "patient portal*"
11. "telemedicine"
12. "mhealth"
13. "ehealth"
14. "telehealth"
15. "mobile health"
16. "hospital information system*"
17. "computerized provider order entry"
18. "closed loop medic*""
19. "nursing informatic*"
20. "nursing information system*"
21. "interoperab*"
22. "consumer health informatic*"
23. "cdss"
24. "cpoe"
25. "ehr"
26. "emr"
27. "artificial intelligence"
28. "blockchain*"
29. "distributed ledger*"
30. "e-health"
31. "radiology information system*"
32. "picture archiving and communication system*"
33. "pacs"
34. OR/1-33
35. "quality improvement*"
36. "meaningful use"
37. "quality of health care”
38. "quality of healthcare”
39. "health care quality”
40. "healthcare quality”
41. "quality of care”
42. "care quality”
43. "patient outcome*”
44. "treatment outcome*”
45. "patient relevant outcome*”
46. "effectiveness”
47. "efficiency”
48. "productivity”
49. "cost-effectiveness”
50. "cost effectiveness”
51. "cost benefit*”
52. "cost-benefit*”
53. "cost saving*”
54. "patient safety”
55. "medical error*”
56. "medication error*”
57. "prescription error*”
58. "patient acceptance of health care”
59. "health care utilization”
60. "healthcare utilization”
61. "patient compliance”
62. "medication adherence”
63. "drug adherence”
64. "medication compliance”
65. "patient participation”
66. "patient engagement”
67. "digital divide”
68. "health equity”
69. "learning health system*”
70. "access to health”
71. "accessibility”
72. "prescribing error*”
73. "unintended consequence*”
74. "adverse effect*”
75. "outcome*”
76. OR/35-75
77. 34 AND 76
78. 77 AND Date range 11/2011 – 11/2023
