# Supplementary material for: Evaluating Methodological Coherence and Evidence Recognition in Digital Health Systematic Reviews: Sample-based Meta-research Study
Source: Online J Public Health Inform. 2026 Apr 16;18:e78210. doi: 10.2196/78210 (PMC13086262; doi:10.2196/78210)
Supplement: Multimedia Appendix 2 [file ojphi-v18-e78210-s002.docx]

# Multimedia Appendix 2

## Inclusion criteria

1. MUST: Systematic review: It must be a systematic review and, if applicable, a meta-analysis or (systematic) umbrella review or overview of systematic reviews.
   Protocols of Systematic Reviews, non-systematic reviews, Rapid Reviews, Realist Reviews and Scoping Reviews are excluded; exception: Systematic Scoping Review and Meta-Analysis.
   NB: Integrative reviews are a special form that is always decided on a case-by-case basis! Integrative reviews are only included if they report on the relationship between relevant interventions and outcomes and excluded if the theoretical part focusing on new concepts or frameworks predominates.
2. MUST: Formal quality: The abstract must include key components of a good summary, i.e. aim/purpose/objective, method, intervention (technology), population/setting, outcomes/results, discussion and conclusion (at best structured by headings or clearly visible from the text).
3. MUST: Digital health intervention: The effect of one or more information technologies must be considered in terms of a digital health intervention.
4. MUST: Outcome: The abstract must report on at least one relevant outcome for care seekers and/or caregivers that is directly associated with a digital health intervention.
5. CAN: Future technologies: studies on artificial intelligence, deep/machine learning, NLP, conversational bots, wearables, robotics, blockchain, etc. can be included provided that relevant outcomes of practical application are considered or reported. NB: excluded are studies that do not consider or report relevant outcomes, but merely consider the state of the art, technological methods or the quality of the underlying algorithms/theories, (potential) clinical application areas or challenges in practical implementation.
6. CAN: Setting: One or more clinical settings for humans is considered.
7. CAN: Influencing factors: In addition to outcome(s), factors influencing the achievement of these outcome(s) or the implementation, adoption and use of information technologies are examined.
8. CAN: Study design: Information is provided on the study designs of the primary studies analysed (e.g. RCT).
9. CAN: Effects: Effect sizes are reported.
10. CAN: Time periods: Time periods for the occurrence of effects (technology on outcome) are reported.

## Exclusion criteria

1. None of the 4 MUST inclusion criteria apply
2. Wrong study design: Protocols of Systematic Reviews, non-systematic reviews, Rapid Reviews, Realist Reviews and Scoping Reviews are excluded; exception: Systematic Scoping Review and Meta-Analysis.
3. Poor formal quality: The abstract is unstructured, very brief, plain text and does not include clear information on intervention (technology) and/or outcomes/results.
4. Wrong intervention or technology: Medical technologies or procedure techniques without focus on digital health interventions, e.g., ultrasound, point of care testing, imaging machines (e.g. CT, MRI), radiomics, colonoscopies, endoscopic resection techniques, etc.
5. Wrong intervention focus: Several interventions are analyzed, of which only a minor part (<50%) contains a digital health component.
   The interventions and outcomes are primarily aimed at education.
   The intervention under investigation is primarily aimed at research-relevant outcomes, e.g. patient recruitment, forming cohorts, conducting RCTs and other studies.
6. Wrong outcome(s): studies that primarily investigate and present the implementation, adoption, acceptance, engagement, experiences, usability, perceptions and/or use of the technologies, or barriers and facilitators, success factors or strategies relating to these.
   Studies that analyze, present or develop frameworks, e.g. health assessment frameworks, conceptual frameworks, decision making frameworks.
