# Supplementary material for: Evaluating Methodological Coherence and Evidence Recognition in Digital Health Systematic Reviews: Sample-based Meta-research Study
Source: Online J Public Health Inform. 2026 Apr 16;18:e78210. doi: 10.2196/78210 (PMC13086262; doi:10.2196/78210)
Supplement: Multimedia Appendix 3 [file ojphi-v18-e78210-s003.docx]

# Multimedia Appendix 3

Table S1. Systematic classification of the level of specification of PICO-elements.

| **PICO-Elements** | **Classification for the Quantitative Analysis** | **Examples of Extracted Data** |
| --- | --- | --- |
| P 1 - Problem - Disorder/Condition - as specified in Title and/or Objectives and/or Methods | High - one medical problem (e.g., disease/disorder/condition) or objective is clearly specified | e.g., acute ischemic stroke [1]; Diabetes-Related Foot Disease [2]; Reducing blood pressure [3]; Sickle Cell Disease [4]; smoking cessation [5] |
|  | Medium - the research covers a relatively homogenous group of medical problems or objectives, often related to one specialty | e.g., Cancer [6]; Cardiovascular Diseases (CVD) [7]; musculoskeletal conditions [8]; Neurorehabilitation [9]; Palliative Care [10]; rheumatology [11]; urology [12] |
|  | Low - the research covers multiple highly heterogenous medical problems or objectives related to multiple medical specialties | e.g., aged care [13]; chronic conditions [14]; Cognitive Impairment [15]; Disabilities [16]; mental health issues [17]; Noncommunicable Diseases [18] |
|  | Not specified |  |
| P 2 – Population - User Group - as specified in Title and/or Objectives and/or Methods | High - the population or target group is clearly specified (e.g., by disorder/condition AND additional characteristics such as age) | e.g., healthy young adults (aged 18-35 years) [19]; Rural Dementia Caregivers [20]; young kidney transplant recipients aged 16 to 30 years [21] |
|  | Medium - the research covers a relatively homogenous population or target group (e.g., patients OR caregivers OR providers OR patient-provider interaction by disorder/condition or medical specialty) | e.g., clinician … in the intensive care unit ; diabetes patients [22]; individuals with heart failure (HF) [23]; Patients Undergoing Cardiac Surgery [24]; patients with HIV [25]; Women with Breast Cancer [26] |
|  | Low - the research covers one or more highly heterogenous populations or target groups (e.g., children and adults, patients and providers) | e.g., clinical practice [27]; displaced populations [14]; Health Care Workers [28]; older adults [29]; Older Persons [30]; young adults [31] |
|  | Not specified |  |
| I - Intervention - Technology - as specified in Title and/or Objectives and/or Methods | High - research focuses on one specific technology or aspect within a homogenous group of technologies | e.g., Artificial Intelligence Methods in Medical Imaging [32]; Cardiac Telerehabilitation [33]; chemotherapy prescription clinical decision-support systems [34]; Electronic medication reconciliation [35]; Telemonitoring [36]; Telepsychiatry [37] |
|  | Medium - research focuses on a relatively homogenous group of technologies | e.g., Telemedicine [21–28][1,22,38–41]; Telerehabilitation [8,42]; mHealth [7,9,15,28,43,43,44]; electronic health records [14,45]; Interventions to reduce medication errors [46]; Clinical Decision Support Systems [47] |
|  | Low - research covers an unspecific range of highly heterogenous technologies | e.g., Digital health technologies [48]; Digital interventions [16,49]; eHealth [5,50]; Healthcare Technologies [30]; Mobile Phone Technologies [51]; remote interventions [52] |
|  | Not specified |  |

Table S1 (continued). Systematic classification of the level of specification of PICO-elements.

| **PICO-Elements** | **Classification for the Quantitative Analysis** | **Examples of Extracted Data** |
| --- | --- | --- |
| C - Comparison – comparable clinical practice or setting as specified anywhere in the Title or Abstract | High - research focuses on one specific and specified care setting, to which the intervention compares and/or applies | e.g., Compared with Traditional {Neurological} Care [39]; intensive care unit [53]; versus face-to-face rehabilitation [8] |
|  | Medium - research compares and/or applies to a specified and relatively homogenous group of care settings or explicitly focuses on intersectoral exchange | e.g., hospital [35,54,55]; Health Information Exchange [56,57]; Home Health Care [58]; Outpatient Setting [59]; Primary Care [6]; self-care [7,29,60] |
|  | Low - research compares and/or applies to an unspecific range of highly heterogenous care practices or settings, which may include no care, self-care and multiple healthcare settings without focus on exchange | e.g., Clinical Settings [61]; Compared to nonactive control groups (eg, usual care) [5]; follow-up care [40]; non-exercising comparator and exercising comparator [62]; Usual Care [22,23,36,41,63–65] |
|  | Not specified |  |
| O - Outcome - as specified in Title and/or Objectives and/or Methods | High - research focuses on one specific and specified outcome with defined indicators | e.g., adherence to ART [25]; detection of commonly missed polyps [66]; Diagnostic Accuracy … for Pulmonary Tuberculosis [32]; Reducing blood pressure [3]; reduce medication errors [46] |
|  | Medium - research evaluates one specified and relatively homogenous group of outcomes or a small number of specified and predefined but diverse outcomes of interest (e.g., mortality, hospitalization, and emergency department visits) | e.g., cost-effectiveness [48,67]; Effects on Cognitive Function and Activities of Daily Living [68]; effectiveness of exercise interventions [69]; Impact on Gait and Dynamic Balance Outcomes [9]; pregnancy outcomes [28]; weight management outcomes [19] |
|  | Low - research has no outcome focus, but aims to identify/evaluate a range of heterogenous outcomes, which are not specified or predefined as primary and secondary outcomes of interest | e.g., Effects ... on the chemotherapy process [34]; effect … on self-care improvement [29]; Categorizing Health Outcomes and Efficacy [15]; Impact ... on Outcomes for Patients [70] |
|  | Not specified - the research has no defined focus on outcomes, but investigates outcomes as one of multiple objectives | e.g., assess the geography of sample populations, scope of interventions, and study outcomes [20]; clinical, fiscal and environmental evidence [12] |

Table S2. Systematic classification of the likelihood of conclusive evidence.

| **Likelihood of Conclusive Evidence** | **Criteria for Classification and Study Selection** | **Examples of Extracted Data** |
| --- | --- | --- |
| High | Meta-Analysis AND multiple (>1) “significant” results reported | e.g., A Meta-Analysis of Randomized Controlled Trials AND significantly improved wound healing rate (RR = 1.44, 95% CI = 1.16-1.80, p = 0.001) and reduced adverse events (RR = 0.52, 95% CI = 0.34-0.80, p = 0.003). [41] |
| Medium | Meta-Analysis AND at least comparable results to usual care OR no meta-analysis, but at least one “significant” effect reported for >25% of relevant studies | e.g., meta-analyses demonstrating long-term ulcer healing and mortality were not significantly different between telehealth and standard care groups [2] |
| Low | Neither meta-analysis nor any “significant” results reported. Evidence is mainly or only reported qualitatively. If any quantitative results are reported, none is described as “significant”. | e.g., Findings demonstrated limited evidence supporting the effectiveness of exercise delivered via telerehabilitation [69] |
| Inconclusive | Evidence is not reported at all OR without any directional conclusion OR although studies report positive evidence, also quantitatively, there are equally contradictive effects reported. | e.g., Authors' conclusions There is no clear good quality evidence for or against using virtual reality … [71]; There is insufficient evidence in the existing literature concerning the real impact of AI or DSS … [55]; Four of the studies indicated that electronic prescribing significantly increases initial medication adherence, while four of the studies suggested the opposite. The remaining two studies found no significant difference …[59] |

## References

1. Zhai Y, Zhu W, Hou H, Sun D, Zhao J. Efficacy of telemedicine for thrombolytic therapy in acute ischemic stroke: a meta-analysis. J Telemed Telecare 2015 Apr;21(3):123–130. doi: 10.1177/1357633X15571357

2. Drovandi A, Wong S, Seng L, Crowley B, Alahakoon C, Banwait J, Fernando ME, Golledge J. Remotely Delivered Monitoring and Management of Diabetes-Related Foot Disease: An Overview of Systematic Reviews. J Diabetes Sci Technol 2023 Jan;17(1):59–69. doi: 10.1177/19322968211012456

3. Liu S, Dunford SD, Leung YW, Brooks D, Thomas SG, Eysenbach G, Nolan RP. Reducing Blood Pressure With Internet-Based Interventions: A Meta-analysis. Can J Cardiol 2013 May;29(5):613–621. doi: 10.1016/j.cjca.2013.02.007

4. Badawy SM, Cronin RM, Hankins J, Crosby L, DeBaun M, Thompson AA, Shah N. Patient-Centered eHealth Interventions for Children, Adolescents, and Adults With Sickle Cell Disease: Systematic Review. J Med Internet Res 2018 Jul 19;20(7):e10940. doi: 10.2196/10940

5. Do H, Tran B, Pham Q, Nguyen L, Tran T, Latkin C, Dunne MP, Baker P. Which eHealth interventions are most effective for smoking cessation? A systematic review. Patient Prefer Adherence 2018 Oct;Volume 12:2065–2084. doi: 10.2147/PPA.S169397

6. Jones OT, Calanzani N, Saji S, Duffy SW, Emery J, Hamilton W, Singh H, De Wit NJ, Walter FM. Artificial Intelligence Techniques That May Be Applied to Primary Care Data to Facilitate Earlier Diagnosis of Cancer: Systematic Review. J Med Internet Res 2021 Mar 3;23(3):e23483. doi: 10.2196/23483

7. Adawiah, Tee, J.N., Johan, M.R.M., Zuhdi, A.S.M., Rafik-Galea, S., Anuar, H. Mhealth in Cardiovascular Diseases (CVD) Self-care: A Systematic Review of Advantages and Challenges. Malays J Med Health Sci 2021;17:129–157. Available from: https://medic.upm.edu.my/upload/dokumen/2021112319003119)_2021_0266.pdf [accessed Feb 29, 2024]

8. Krzyzaniak N, Cardona M, Peiris R, Michaleff ZA, Greenwood H, Clark J, Scott AM, Glasziou P. Telerehabilitation versus face-to-face rehabilitation in the management of musculoskeletal conditions: a systematic review and meta-analysis. Phys Ther Rev 2023 Mar 4;28(2):71–87. doi: 10.1080/10833196.2023.2195214

9. Moreno-Ligero M, Lucena-Anton D, Salazar A, Failde I, Moral-Munoz JA. mHealth Impact on Gait and Dynamic Balance Outcomes in Neurorehabilitation: Systematic Review and Meta-analysis. J Med Syst 2023 Jul 18;47(1):75. doi: 10.1007/s10916-023-01963-y

10. Head BA, Schapmire TJ, Zheng Y. Telehealth in Palliative Care: A Systematic Review of Patient-Reported Outcomes. J Hosp Palliat Nurs 2017 Apr;19(2):130–139. doi: 10.1097/NJH.0000000000000319

11. McDougall JA, Ferucci ED, Glover J, Fraenkel L. Telerheumatology: A Systematic Review. Arthritis Care Res 2017 Oct;69(10):1546–1557. doi: 10.1002/acr.23153

12. Edison MA, Connor MJ, Miah S, El‐Husseiny T, Winkler M, Dasgupta R, Ahmed HU, Hrouda D. Understanding virtual urology clinics: a systematic review. BJU Int 2020 Nov;126(5):536–546. doi: 10.1111/bju.15125

13. Seaman K, Ludlow K, Wabe N, Dodds L, Siette J, Nguyen A, Jorgensen M, Lord SR, Close JCT, O’Toole L, Lin C, Eymael A, Westbrook J. The use of predictive fall models for older adults receiving aged care, using routinely collected electronic health record data: a systematic review. BMC Geriatr 2022 Dec;22(1):210. doi: 10.1186/s12877-022-02901-2

14. Buford A, Ashworth HC, Ezzeddine FL, Dada S, Nguyen E, Ebrahim S, Zhang A, Lebovic J, Hamvas L, Prokop LJ, Midani S, Chilazi M, Alahdab F. Systematic review of electronic health records to manage chronic conditions among displaced populations. BMJ Open 2022 Sep;12(9):e056987. doi: 10.1136/bmjopen-2021-056987

15. Bateman DR, Srinivas B, Emmett TW, Schleyer TK, Holden RJ, Hendrie HC, Callahan CM. Categorizing Health Outcomes and Efficacy of mHealth Apps for Persons With Cognitive Impairment: A Systematic Review. J Med Internet Res 2017 Aug 30;19(8):e301. doi: 10.2196/jmir.7814

16. Zhou L, Parmanto B. Reaching People With Disabilities in Underserved Areas Through Digital Interventions: Systematic Review. J Med Internet Res 2019 Oct 25;21(10):e12981. doi: 10.2196/12981

17. Falconer E, Kho D, Docherty JP. Use of technology for care coordination initiatives for patients with mental health issues: a systematic literature review. Neuropsychiatr Dis Treat 2018 Sep;Volume 14:2337–2349. doi: 10.2147/NDT.S172810

18. Lunde P, Nilsson BB, Bergland A, Kværner KJ, Bye A. The Effectiveness of Smartphone Apps for Lifestyle Improvement in Noncommunicable Diseases: Systematic Review and Meta-Analyses. J Med Internet Res 2018 May 4;20(5):e162. doi: 10.2196/jmir.9751

19. Willmott TJ, Pang B, Rundle-Thiele S, Badejo A. Weight Management in Young Adults: Systematic Review of Electronic Health Intervention Components and Outcomes. J Med Internet Res 2019 Feb 6;21(2):e10265. doi: 10.2196/10265

20. Ruggiano N, Brown EL, Li J, Scaccianoce M. Rural Dementia Caregivers and Technology: What Is the Evidence? Res Gerontol Nurs 2018 Jul;11(4):216–224. doi: 10.3928/19404921-20180628-04

21. Bul KCM, Bannon C, Krishnan N, Dunlop A, Szczepura A. Can eHealth applications improve renal transplant outcomes for adolescents and young adults? A systematic review. Transplant Rev 2023 Apr;37(2):100760. doi: 10.1016/j.trre.2023.100760

22. Hu Y, Wen X, Wang F, Yang D, Liu S, Li P, Xu J. Effect of telemedicine intervention on hypoglycaemia in diabetes patients: A systematic review and meta-analysis of randomised controlled trials. J Telemed Telecare 2019 Aug;25(7):402–413. doi: 10.1177/1357633X18776823

23. Kotb A, Cameron C, Hsieh S, Wells G. Comparative Effectiveness of Different Forms of Telemedicine for Individuals with Heart Failure (HF): A Systematic Review and Network Meta-Analysis. Wu W-CH, editor. PLOS ONE 2015 Feb 25;10(2):e0118681. doi: 10.1371/journal.pone.0118681

24. Wu KA, Kunte S, Rajkumar S, Venkatraman V, Kim G, Kaplan S, Anwar-Hashmi SO, Doberne J, Nguyen TC, Lad SP. Digital Health for Patients Undergoing Cardiac Surgery: A Systematic Review. Healthcare 2023 Aug 28;11(17):2411. doi: 10.3390/healthcare11172411

25. Esmaeili ED, Azizi H, Dastgiri S, Kalankesh LR. Does telehealth affect the adherence to ART among patients with HIV? A systematic review and meta-analysis. BMC Infect Dis 2023 Mar 17;23(1):169. doi: 10.1186/s12879-023-08119-w

26. Tokgöz P, Dockweiler C. Telemedizin in der rehabilitativen Nachsorge von Frauen mit Brustkrebs. Tumordiagn Ther 2023 Jun;44(05):341–348. doi: 10.1055/a-2078-1718

27. Mickan S, Atherton H, Roberts NW, Heneghan C, Tilson JK. Use of handheld computers in clinical practice: a systematic review. BMC Med Inform Decis Mak 2014 Dec;14(1):56. doi: 10.1186/1472-6947-14-56

28. Amoakoh-Coleman M, Borgstein AB-J, Sondaal SF, Grobbee DE, Miltenburg AS, Verwijs M, Ansah EK, Browne JL, Klipstein-Grobusch K. Effectiveness of mHealth Interventions Targeting Health Care Workers to Improve Pregnancy Outcomes in Low- and Middle-Income Countries: A Systematic Review. J Med Internet Res 2016 Aug 19;18(8):e226. doi: 10.2196/jmir.5533

29. Rostam Niakan Kalhori S, Rahmani Katigari M, Talebi Azadboni T, Pahlevanynejad S, Hosseini Eshpala R. The effect of m-health applications on self-care improvement in older adults: A systematic review. Inform Health Soc Care 2023 Jul 3;48(3):292–331. doi: 10.1080/17538157.2023.2171878

30. Vasquez BA, Betriana F, Nemenzo E, Inabangan AK, Tanioka R, Garcia L, Juntasopeepun P, Tanioka T, Locsin RC. Effects of Healthcare Technologies on the Promotion of Physical Activities in Older Persons: A Systematic Review. Inform Health Soc Care 2023 Apr 3;48(2):196–210. doi: 10.1080/17538157.2022.2086874

31. Oosterveen E, Tzelepis F, Ashton L, Hutchesson MJ. A systematic review of eHealth behavioral interventions targeting smoking, nutrition, alcohol, physical activity and/or obesity for young adults. Prev Med 2017 Jun;99:197–206. doi: 10.1016/j.ypmed.2017.01.009

32. Zhan Y, Wang Y, Zhang W, Ying B, Wang C. Diagnostic Accuracy of the Artificial Intelligence Methods in Medical Imaging for Pulmonary Tuberculosis: A Systematic Review and Meta-Analysis. J Clin Med 2022 Dec 30;12(1):303. doi: 10.3390/jcm12010303

33. Zhong W, Liu R, Cheng H, Xu L, Wang L, He C, Wei Q. Longer-Term Effects of Cardiac Telerehabilitation on Patients With Coronary Artery Disease: Systematic Review and Meta-Analysis. JMIR MHealth UHealth 2023 Jul 28;11:e46359. doi: 10.2196/46359

34. Rahimi R, Moghaddasi H, Rafsanjani KA, Bahoush G, Kazemi A. Effects of chemotherapy prescription clinical decision-support systems on the chemotherapy process: A systematic review. Int J Med Inf 2019 Feb;122:20–26. doi: 10.1016/j.ijmedinf.2018.11.004

35. Wang H, Meng L, Song J, Yang J, Li J, Qiu F. Electronic medication reconciliation in hospitals: a systematic review and meta-analysis. Eur J Hosp Pharm 2018 Sep;25(5):245–250. doi: 10.1136/ejhpharm-2017-001441

36. Yun JE, Park J-E, Park H-Y, Lee H-Y, Park D-A. Comparative Effectiveness of Telemonitoring Versus Usual Care for Heart Failure: A Systematic Review and Meta-analysis. J Card Fail 2018 Jan;24(1):19–28. doi: 10.1016/j.cardfail.2017.09.006

37. Hassan A, Sharif K. Efficacy of Telepsychiatry in Refugee Populations: A Systematic Review of the Evidence. Cureus 2019 Jan 30; doi: 10.7759/cureus.3984

38. Shah AC, O’Dwyer LC, Badawy SM. Telemedicine in Malignant and Nonmalignant Hematology: Systematic Review of Pediatric and Adult Studies. JMIR MHealth UHealth 2021 Jul 8;9(7):e29619. doi: 10.2196/29619

39. Maida E, Bresciamorra V, Triassi M, Lanzillo R, Bonavita S, Lavorgna L. Cost-Analysis of Telemedicine Interventions Compared with Traditional Care in the Management of Chronic Neurological Diseases: A Systematic Review. Telemed E-Health 2024 Feb 1;30(2):404–414. doi: 10.1089/tmj.2023.0106

40. Nordheim LV, Haavind MT, Iversen MM. Effect of telemedicine follow-up care of leg and foot ulcers: a systematic review. BMC Health Serv Res 2014 Dec;14(1):565. doi: 10.1186/s12913-014-0565-6

41. Huang Z, Wu S, Yu T, Hu A. Efficacy of Telemedicine for Patients with Chronic Wounds: A Meta-Analysis of Randomized Controlled Trials. Adv Wound Care 2021 Feb 1;10(2):103–112. doi: 10.1089/wound.2020.1169

42. Martínez-Guijarro C, López-Fernández MD, Lopez-Garzon M, Lozano-Lozano M, Arroyo-Morales M, Galiano-Castillo N. Feasibility and efficacy of telerehabilitation in the management of patients with head and neck cancer during and after oncological treatment: A systematic review. Eur J Oncol Nurs 2023 Apr;63:102279. doi: 10.1016/j.ejon.2023.102279

43. Mokaya M, Kyallo F, Vangoitsenhoven R, Matthys C. Clinical and patient-centered implementation outcomes of mHealth interventions for type 2 diabetes in low-and-middle income countries: a systematic review. Int J Behav Nutr Phys Act 2022 Dec;19(1):1. doi: 10.1186/s12966-021-01238-0

44. Wang L, Langlais CS, Kenfield SA, Chan JM, Graff RE, Allen IE, Atreya CE, Van Blarigan EL. mHealth Interventions to Promote a Healthy Diet and Physical Activity among Cancer Survivors: A Systematic Review of Randomized Controlled Trials. Cancers 2022 Aug 6;14(15):3816. doi: 10.3390/cancers14153816

45. Baer HJ, Cho I, Walmer RA, Bain PA, Bates DW. Using Electronic Health Records to Address Overweight and Obesity. Am J Prev Med 2013 Oct;45(4):494–500. doi: 10.1016/j.amepre.2013.05.015

46. Manias E, Kusljic S, Wu A. Interventions to reduce medication errors in adult medical and surgical settings: a systematic review. Ther Adv Drug Saf 2020 Jan;11:204209862096830. doi: 10.1177/2042098620968309

47. Blum D, Raj SX, Oberholzer R, Riphagen II, Strasser F, Kaasa S. Computer-Based Clinical Decision Support Systems and Patient-Reported Outcomes: A Systematic Review. Patient - Patient-Centered Outcomes Res 2015 Oct;8(5):397–409. doi: 10.1007/s40271-014-0100-1

48. Puleo V, Gentili A, Failla G, Melnyk A, Di Tanna G, Ricciardi W, Cascini F. Digital health technologies: a systematic review of their cost-effectiveness. Eur J Public Health 2021 Oct 1;31(Supplement_3):ckab164.273. doi: 10.1093/eurpub/ckab164.273

49. Martinez Agulleiro L, Patil B, Firth J, Sawyer C, Amann BL, Fonseca F, Torrens M, Perez V, Castellanos FX, Kane JM, Guinart D. A systematic review of digital interventions for smoking cessation in patients with serious mental illness. Psychol Med 2023 Aug;53(11):4856–4868. doi: 10.1017/S003329172300123X

50. Bui LK, Park M, Giap T-T-T. eHealth interventions for the informal caregivers of people with dementia: A systematic review of systematic reviews. Geriatr Nur (Lond) 2022 Nov;48:203–213. doi: 10.1016/j.gerinurse.2022.09.015

51. Indraratna P, Tardo D, Yu J, Delbaere K, Brodie M, Lovell N, Ooi S-Y. Mobile Phone Technologies in the Management of Ischemic Heart Disease, Heart Failure, and Hypertension: Systematic Review and Meta-Analysis. JMIR MHealth UHealth 2020 Jul 6;8(7):e16695. doi: 10.2196/16695

52. Suntai Z, Laha-Walsh K, Albright DL. Effectiveness of remote interventions in improving caregiver stress outcomes for caregivers of people with traumatic brain injury. Rehabil Psychol 2021 Nov;66(4):415–422. doi: 10.1037/rep0000402

53. Hains IM, Georgiou A, Westbrook JI. The impact of PACS on clinician work practices in the intensive care unit: a systematic review of the literature. J Am Med Inform Assoc 2012 Jul 1;19(4):506–513. doi: 10.1136/amiajnl-2011-000422

54. Veldhuis LI, Woittiez NJC, Nanayakkara PWB, Ludikhuize J. Artificial Intelligence for the Prediction of In-Hospital Clinical Deterioration: A Systematic Review. Crit Care Explor 2022 Aug 26;4(9):e0744. doi: 10.1097/CCE.0000000000000744

55. Toffaha KM, Simsekler MCE, Omar MA. Leveraging artificial intelligence and decision support systems in hospital-acquired pressure injuries prediction: A comprehensive review. Artif Intell Med 2023 Jul;141:102560. doi: 10.1016/j.artmed.2023.102560

56. Bloomrosen M, Berner ES. Findings from the 2022 Yearbook Section on Health Information Exchange. Yearb Med Inform 2022 Aug;31(01):215–218. doi: 10.1055/s-0042-1742534

57. Dobrow MJ, Bytautas JP, Tharmalingam S, Hagens S. Interoperable Electronic Health Records and Health Information Exchanges: Systematic Review. JMIR Med Inform 2019 Jun 6;7(2):e12607. doi: 10.2196/12607

58. Mun M, Park Y, Hwang J, Woo K. Types and Effects of Telenursing in Home Health Care: A Systematic Review and Meta-Analysis. Telemed E-Health 2023 Sep 14;tmj.2023.0188. doi: 10.1089/tmj.2023.0188

59. Aluga D, Nnyanzi LA, King N, Okolie EA, Raby P. Effect of Electronic Prescribing Compared to Paper-Based (Handwritten) Prescribing on Primary Medication Adherence in an Outpatient Setting: A Systematic Review. Appl Clin Inform 2021 Aug;12(04):845–855. doi: 10.1055/s-0041-1735182

60. Alsayed AO, Ismail NA, Hasan L, Syed AH, Embarak F, Da’u A. A systematic literature review for understanding the effectiveness of advanced techniques in diabetes self-care management. Alex Eng J 2023 Sep;79:274–295. doi: 10.1016/j.aej.2023.08.026

61. Yan AF, Chen Z, Wang Y, Campbell JA, Xue Q-L, Williams MY, Weinhardt LS, Egede LE. Effectiveness of Social Needs Screening and Interventions in Clinical Settings on Utilization, Cost, and Clinical Outcomes: A Systematic Review. Health Equity 2022 Jun 1;6(1):454–475. doi: 10.1089/heq.2022.0010

62. Brown RC, Coombes JS, Jungbluth Rodriguez K, Hickman IJ, Keating SE. Effectiveness of exercise via telehealth for chronic disease: a systematic review and meta-analysis of exercise interventions delivered via videoconferencing. Br J Sports Med 2022 Sep;56(18):1042–1052. doi: 10.1136/bjsports-2021-105118

63. Tavares Franquez R, Del Grossi Moura M, Cristina Ferreira McClung D, Barberato-Filho S, Cruz Lopes L, Silva MT, De Sá Del-Fiol F, De Cássia Bergamaschi C. E-Health technologies for treatment of depression, anxiety and emotional distress in person with diabetes mellitus: A systematic review and meta-analysis. Diabetes Res Clin Pract 2023 Sep;203:110854. doi: 10.1016/j.diabres.2023.110854

64. Faiçal AVB, Mota LR, Correia D d’ A, Monteiro LP, Souza EL de, Terse-Ramos R. Telehealth for children and adolescents with chronic pulmonary disease: systematic review. Rev Paul Pediatr Orgao Of Soc Pediatr Sao Paulo 2023;42:e2024111. PMID:37194911

65. Karataş N, Kaya A, İşler Dalgıç A. The effectiveness of user-focused mobile health applications in paediatric chronic disease management: A systematic review. J Pediatr Nurs 2022 Mar;63:e149–e156. doi: 10.1016/j.pedn.2021.09.018

66. Sivananthan A, Nazarian S, Ayaru L, Patel K, Ashrafian H, Darzi A, Patel N. Does computer-aided diagnostic endoscopy improve the detection of commonly missed polyps? A meta-analysis. Clin Endosc 2022 May 30;55(3):355–364. doi: 10.5946/ce.2021.228

67. Law L, Kelly JT, Savill H, Wallen MP, Hickman IJ, Erku D, Mayr HL. Cost-effectiveness of telehealth-delivered diet and exercise interventions: A systematic review. J Telemed Telecare 2022 Feb 2;1357633X2110707. doi: 10.1177/1357633X211070721

68. Chen X, Liu F, Lin S, Yu L, Lin R. Effects of Virtual Reality Rehabilitation Training on Cognitive Function and Activities of Daily Living of Patients With Poststroke Cognitive Impairment: A Systematic Review and Meta-Analysis. Arch Phys Med Rehabil 2022 Jul;103(7):1422–1435. doi: 10.1016/j.apmr.2022.03.012

69. Leslie S, Tan J, McRae PJ, O’Leary SP, Adsett JA. The Effectiveness of Exercise Interventions Supported by Telerehabilitation For Recently Hospitalized Adult Medical Patients: A Systematic Review. Int J Telerehabilitation 2021 Dec 15;13(2). doi: 10.5195/ijt.2021.6356

70. Shah S, Yeheskel A, Hossain A, Kerr J, Young K, Shakik S, Nichols J, Yu C. The Impact of Guideline Integration into Electronic Medical Records on Outcomes for Patients with Diabetes: A Systematic Review. Am J Med 2021 Aug;134(8):952-962.e4. doi: 10.1016/j.amjmed.2021.03.004

71. Välimäki M, Hätönen HM, Lahti ME, Kurki M, Hottinen A, Metsäranta K, Riihimäki T, Adams CE. Virtual reality for treatment compliance for people with serious mental illness. Cochrane Schizophrenia Group, editor. Cochrane Database Syst Rev 2014 Oct 8; doi: 10.1002/14651858.CD009928.pub2
